# Exploring Conversational Dynamics in Scientific and Pseudoscientific Health Communities on YouTube: Process Mining and Network Analysis Study

**DOI:** 10.2196/95273

**Published:** 2026-10-02

**Authors:** Stefan Anca, Samuel Paul Gallegos-Serrano, Carlos Luis Sánchez-Bocanegra, Paolo Piraino, José Luis Sevillano Ramos

**Affiliations:** 1 Organon SRL, Bucharest, Romania; 2 Estudios de Ciencias de la Salud, Universitat Oberta de Catalunya, Barcelona, Spain; 3 ETS Ingenieria Informatica, Universidad de Sevilla, Avenida Reina Mercedes, s/n, Sevilla, Andalusia, 41012, Spain, 34 954556142

**Keywords:** health communication, social media, YouTube, misinformation, pseudoscience, natural language processing, process mining, network analysis, infodemiology

## Abstract

**Background:**

Social media platforms, particularly YouTube (Google LLC), are important sources of health information, but also significant vectors for misinformation and pseudoscience. While many studies analyze the content and sentiment of this information, the dynamic, sequential nature of user interactions, which can shape belief formation and community dynamics, remains poorly understood.

**Objective:**

This study aimed to explore the applicability of network analysis and process mining techniques for identifying and comparing structural and emotional patterns of conversational flow within a retrieved corpus of YouTube comments from videos about scientific or pseudoscientific health treatments.

**Methods:**

We conducted an exploratory observational study using publicly available YouTube comment threads posted between 2011 and 2025 from videos categorized as either “scientific” (20,387 comments) or “pseudoscientific” (32,025 comments) using an automated pipeline that combined API-based data extraction, large language model–based video classification, and natural language processing techniques for multilingual sentiment and thematic classification of comments. We then applied process mining to model the temporal sequences of interactions, and network analysis to map the relationships between conversational topics.

**Results:**

Network analysis revealed divergent conversational cores. In the scientific corpus, negative expressions of feelings and negative comparisons had greater normalized node strength, while medical treatment and advice requests were also relatively more prominent. In the pseudoscientific corpus, positive expressions of feelings, thanking, compliments, and emoji-only or brief acknowledgments showed greater prominence. Process mining showed a more heterogeneous combination of negative, positive, and neutral activities in the scientific corpus, whereas the most frequent pathways in the pseudoscientific corpus were concentrated around positive expressions of feelings, thanking, compliments, and brief messages of acknowledgment, or simply emojis.

**Conclusions:**

The application of network analysis and process mining techniques revealed distinct patterns in the conversational dynamics of health-related YouTube discussions. Within the sampled corpora, scientific discussions appeared more compatible with mixed-valence evaluation and treatment-related exchanges, whereas pseudoscientific discussions showed patterns more consistent with interpersonal affirmation and socioemotional bonding. These preliminary findings suggest that network analysis and process mining are promising approaches for investigating online health communication and misinformation ecosystems. They also suggest that public health strategies may benefit from considering the affective and community-bonding dimensions of engagement in misinformation communities alongside information provision.

## Introduction

### The Proliferation of Health Misinformation on Social Media Platforms

In recent decades, the digital media landscape has enabled the widespread dissemination of pseudoscientific health discourses [1]. Platforms such as YouTube (Google LLC), with more than 2.5 billion monthly users, have become major sources of health information. However, systematic reviews have found that health-related content on YouTube is often of average-to-below-average quality and that unreliable or misleading information remains prevalent across health topics [2-5]. This phenomenon, often termed an “infodemic,” refers to an overabundance of information that makes it difficult to find trustworthy sources and reliable guidance [6]. The COVID-19 pandemic highlighted how social media can amplify unverified information, conspiracy theories, and false claims, with potentially serious consequences for public trust and health [6]. Health misinformation remains a persistent public health challenge beyond the pandemic [7].

Health misinformation is broadly defined as a health-related claim that is false, misleading, or unsupported by current scientific knowledge [8]. Its consequences may include poorer clinical outcomes, reinforcement of false beliefs, and reduced adherence to public health recommendations. For example, among patients with breast cancer, the use of alternative medicine has been associated with shorter survival [9]. It may also reinforce unsupported or illusory health beliefs [10] and reduce adherence to public health guidelines during emergencies [11]. Misinformation is prevalent across topics, including vaccines, smoking products, and noncommunicable diseases such as cancer, with platforms including Twitter (X Corp) and YouTube serving as major venues for these discussions [8].

### Traditional Approaches to Studying Pseudoscientific Communities

Traditional strategies to combat health misinformation, such as fact-checking, have shown limited efficacy. A recent review found that current fact-checking models involve trade-offs between trust, scalability, and impact, with neither professional nor community-based approaches performing strongly across all 3 dimensions [12]. At the individual level, research suggests that users exposed to fact-checking may continue to disseminate incorrect information, indicating that such interventions may be insufficient [13]. Moreover, attempts to debunk science-relevant misinformation may not, on average, produce a statistically significant change in recipients’ beliefs or attitudes, consistent with previous evidence that beliefs can persist despite corrective information, particularly when socially reinforced [14,15]. This suggests the need to better understand the underlying social and psychological drivers of engagement, which can motivate users to conform to group norms [16].

Prior research has identified the existence of polarized online communities. For instance, studies on platforms such as Facebook (Meta Platforms, Inc) have shown that pseudoscientific communities tend to be insular, reinforcing their convictions within echo chambers, whereas scientific communities are more likely to engage with opposing viewpoints in an effort to debunk them [17]. More recent research has continued to identify polarized interaction structures in social media discussions, while also highlighting that network separation does not always map directly onto similarity in expressed opinions [18].

### Limits of Current Interventions and Analyses

To understand these phenomena, research on pseudoscientific discourse has used diverse methods, ranging from manual content classification [19] to network and language analyses. For instance, network analysis has been used to map antivaccine communities on Twitter [20], while natural language processing (NLP) has been used to identify linguistic patterns in denialist discourse [21]. Similarly, text mining of YouTube video titles has been used to track public opinion, although it did not examine user interactions within the comment sections [22]. A recent systematic review of 813 publications distinguished 4 methodological approaches to health misinformation research: quantitative and qualitative social research using participant data, and quantitative and qualitative analyses of digital media data [23]. The review further noted that, although computational approaches are increasingly used to analyze large-scale digital data, they should be complemented by qualitative and traditional social research to provide contextual depth [23]. Another review identified content coding, machine learning, NLP, topic modeling, sentiment analysis, and graph-based approaches to analyze health misinformation across social media platforms [24]. Finally, a framework for social media listening, aimed at understanding public discourse and health-related behaviors, incorporates topic modeling, sentiment analysis, and stance detection [25].

However, methodological diversity at the field level does not necessarily imply integration within individual studies. A systematic review by Suarez-Lledo and Alvarez-Galvez [8] identified substantial fragmentation in this area, with studies commonly focusing on individual approaches such as social network analysis (19/69, 28%) or content evaluation (18/69, 26%). Related work has proposed taxonomies of data science strategies for health misinformation detection, ranging from manual and expert-led assessments to automated approaches [26]. Taken together, these findings suggest that the broad range of available analytical approaches is not yet fully integrated in empirical research. The predominance of single-method studies may limit our understanding of how semantic, structural, and temporal dimensions interact.

As noted by Jain and Achuthan [27], despite extensive work on misinformation spread, existing frameworks often rely on “static models” or basic compartments that overlook adaptive, real-time user interaction. They therefore propose extended models that incorporate behavioral divergence, platform-related factors, and adaptive feedback mechanisms to better approximate real-world propagation dynamics. Moreover, predictive performance alone is insufficient without interpretability. As shown by Xie et al [28], even deep learning models that excel at forecasting health misinformation spread on YouTube may fail to reveal actionable causal drivers due to their black-box nature.

This shift toward modeling fine-grained, context-dependent factors underscores the need to understand the interactive environment underlying misinformation propagation. This aligns with psychological principles of “shared reality theory,” which posit that belief validation is not a static outcome but a dynamic process constructed through interpersonal communication and audience tuning [29]. Moreover, recent frameworks in persuasion psychology, such as the sequential information processing (SIP) model, suggest that the order in which information is received fundamentally alters its interpretation, as initial interactions influence how subsequent ones are processed through mechanisms of assimilation or contrast [30]. Therefore, modeling comment threads as interaction sequences and extracting recurrent pathways can provide a process-level view of conversational reinforcement.

We operationalize these psychological principles by treating comment threads as temporal event logs, from which process mining and network analysis extract recurrent conversational pathways that capture how social reality is constructed through interaction.

### Process Mining and Network Analysis: Novel Methods for Understanding Conversational Dynamics

This study aims to provide this context by introducing a novel methodological framework that combines NLP with process mining and network analysis to analyze conversational dynamics.

Our approach begins by using NLP to characterize each comment according to its thematic content and sentiment [31]. Following this, we apply process mining, a discipline from business process management that discovers, monitors, and improves real-world processes by extracting knowledge from event logs [32]. Process mining has been applied in certain health care contexts, such as clinical pathways and organizational workflows [33]. Process mining has also been applied to social media event data to identify behavioral patterns in online interactions [34] and, more recently, to social network event data to characterize coordinated online behavior and information propagation [35]. In this study, we extend this methodological approach to model the conversational dynamics of health care–related user interactions.

In this approach, a YouTube comment thread is conceptualized as an “event log,” where each comment is an event in a sequence. By applying process mining algorithms to these conversational logs, as described in the general framework by Holstrup and colleagues [32], we can create visual models that map the typical pathways of interaction. This method allows for a direct, data-driven visualization of the “conversational signatures” that characterize different online interactions, which we use here to compare conversational patterns in YouTube discussions associated with scientific and pseudoscientific health content.

As a complement, we integrate a network analysis based on the method of Peeters [36], which studies paired interactions within specific conversations. We build a graph where nodes represent the thematic categories of comments and edges represent the frequency with which one communicative function follows another. This triple combination of NLP, process mining, and network analysis allows us to examine the social interaction process from multiple perspectives, revealing angles that a single method could not observe.

### Study Objectives

The primary objective of this study is to use process mining and thematic network analysis to discover, analyze, visualize, and compare recurrent conversational patterns within the retrieved samples of YouTube comment threads associated with scientific and pseudoscientific health content. A secondary objective is to assess the utility of these complementary methods for characterizing sequential and structural differences in online health-related interactions and for generating hypotheses that may inform future research and communication strategies in the digital information ecosystem.

## Methods

### Reporting Approach

The methodology for this study was structured according to the Cross-Industry Standard Process for Data Mining (CRISP-DM) framework, adapted to fit the standard IMRD (Introduction, Methods, Results, and Discussion) format for a scientific paper [37]. This study was reported following the STROBE (Strengthening the Reporting of Observational Studies in Epidemiology) statement, as the closest applicable guideline for observational research [38]. Given the lack of specific reporting guidance for YouTube-based health research, the checklist was adapted to the characteristics of the study, and nonapplicable items were explicitly identified [39]. The completed STROBE checklist is provided in Checklist 1.

### Data Collection and Corpus Assembly

The search was limited to YouTube because it offers a free, publicly available API that enables straightforward, legal data collection with relatively few access restrictions [40]. Candidate videos were retrieved between May 4 and May 6, 2025, and the associated comment threads were collected between May 5 and May 12, 2025. As both processes partially overlapped, the overall data collection period extended from May 4 to May 12, 2025. The corpus of YouTube comments was compiled using the official YouTube Data API. Data extraction initially intended to focus on videos relevant to Spanish speakers, setting the API parameter relevanceLanguage to “es.” However, the platform’s search algorithms intrinsically retrieve content spanning multiple languages if deemed highly relevant to the query term [41]. Consequently, the resulting corpus exhibited significant linguistic diversity. We decided to retain the multilingual content to capture broader, cross-cultural behavioral patterns.

The search strategy was designed to capture 2 distinct categories of content: scientific medical treatments and pseudoscientific treatments. In this study, “pseudoscientific” is used as an operational analytical category based on established scientific consensus regarding the efficacy of the referenced therapies, and does not constitute a judgment about individual users or their motivations. A set of specific, representative search terms was used for each category, as detailed in Table 1. These terms were initially selected based on prior knowledge of common therapies, followed by a brief web-based verification to confirm whether each term corresponded to a scientifically supported treatment or a pseudoscientific practice. This approach ensured a clear distinction between categories while maintaining practical feasibility for data collection.

To maximize the potential volume of comments for analysis, the API was queried to retrieve the 100 most-viewed videos for each search term that was used. During comment collection, the number of top-level comment threads retrieved per video was capped at 500 to limit the disproportionate contribution of individual videos to the corpus. This approach yielded an initial list of 313 unique videos related to scientific treatments and 474 unique videos related to pseudoscientific treatments. The final analysis was based on a corpus of 20,387 comments from scientific videos and 32,025 comments from pseudoscientific videos.

**Table 1.** Search terms used for data collection.

Terms used to retrieve candidate videos for the scientific-content corpus	Terms used to retrieve candidate videos for the pseudoscientific-content corpus
Tratamiento cáncer (cancer treatment)	Terapia energética (energy therapy)
Procedimiento endoscópico (endoscopic procedure)	Reiki
Terapia cognitivo-conductual (cognitive behavioral therapy)	Naturopatía (naturopathy)
Neurorrehabilitación (neurorehabilitation)	Acupuntura (acupuncture)
Fisioterapia deportiva (sports physiotherapy)	Magnetoterapia (magnet therapy)
Rehabilitación cardíaca (cardiac rehabilitation)	Flores de Bach (Bach flower remedies)

### Video Classification and Filtering

A qualitative classification was performed using Google’s Gemini large language model (LLM). The model was provided with the video’s title, description, and tags and instructed with a detailed prompt to categorize the video as “scientific,” “pseudoscientific,” or “irrelevant.” The prompt explicitly defined each category and instructed the model to default to “irrelevant” if the information was ambiguous or insufficient, ensuring a conservative classification. The full prompt is available in Multimedia Appendix 1.

Only videos where the category of the original search term (scientific or pseudoscientific) matched the LLM’s classification were included. This step was crucial for eliminating ambiguous cases, such as scientific videos debunking pseudoscientific claims that might have been retrieved via a pseudoscientific search term, thereby ensuring a clean separation between the two audience types. The number of videos retrieved from each term, and the volume of comments collected from all the videos of each term, are detailed in Multimedia Appendix 2.

As shown in Figure 1, candidate YouTube videos were retrieved using predefined scientific and pseudoscientific health-treatment search terms, classified as scientific, pseudoscientific, or irrelevant, and filtered before comment collection.

**Figure 1.**
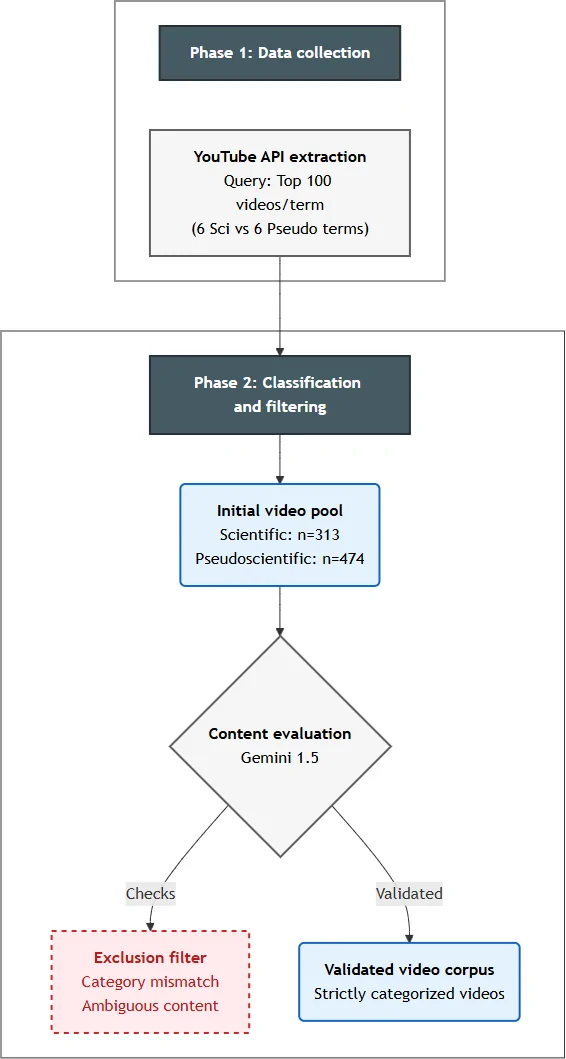
Workflow representation of the process for obtaining the corpus of videos used in the study.

### Comment Feature Engineering

An NLP pipeline was applied to each comment to extract features necessary for the subsequent analyses.

### Conversation Thread Reconstruction

For each top-level (parent) comment, the API was used to retrieve all its direct replies and the parent comment itself. This structure was preserved in the dataset, with each comment linked to its parent and assigned a Conversation_id corresponding to the ID of the initial comment in the thread. Additionally, the timestamp of each comment was recorded to establish the chronological order of events within each conversation. This preservation of sequential information is a prerequisite for applying process mining techniques to the conversations [32].

### Sentiment Analysis

The sentiment of each comment was determined using the nlptown/bert-base-multilingual-uncased-sentiment model from the Hugging Face library [42]. This model provides a score from 1 to 5 stars. For simplicity and clarity in the process models, these scores were mapped to 3 categories: scores of 1 and 2 were classified as “negative,” a score of 3 as “neutral,” and scores of 4 and 5 as “positive.”

### Thematic Categorization

To classify the topic or intent of each comment, a zero-shot classification approach was used using the MoritzLaurer/mDeBERTa-v3-base-xnli-multilingual-nli-2mil7 model from Hugging Face, due to its training in cross-lingual tasks without task-specific fine-tuning, crucial for our multilingual corpus [43,44]. This method enables the classification of text fragments using a predefined set of labels without requiring a model pretrained on those specific labels. For each comment, the model outputs a confidence score for every label in the taxonomy; the label with the highest score was assigned as the definitive category for that comment. The taxonomy of labels was adapted from a comprehensive classification scheme for YouTube comments developed by Madden et al [45]. This choice to build upon an established, peer-reviewed framework lends validity to the categories used.

The taxonomy was simplified by merging related categories, and 3 domain-specific extra categories were added: “medical treatment,” “pseudoscientific treatment,” and “emotional support.” The full, adapted taxonomy is provided in Multimedia Appendix 3. “medical treatment” and “pseudoscientific treatment” were included to verify whether users directly engage with the primary topics that define each group. “emotional support” was included because prior research on online health communities indicates that information sharing is closely associated with the social support received within the community [46].

### Data Quality Control

To ensure the quality of the analytical corpus, several preprocessing steps were implemented. The primary unit of analysis was the conversational thread. Therefore, only comments that were part of a thread containing at least one reply were included; isolated, top-level comments without any replies were not downloaded, as they do not contribute to the conversational structure analyzed in this study. Regarding the linguistic diversity mentioned in the “Data Collection and Corpus Assembly” section, to characterize it, the language of each comment was identified using the “papluca/xlm-roberta-base-language-detection” model from Hugging Face, which supports a wide range of languages [47]. Also, a verification for duplicate comments was performed across the corpus; however, no duplicate entries were identified. For process mining, each conversation was identified by the ID of the top-level comment, and all replies within a thread were ordered chronologically to reconstruct the sequence of interaction.

### Video Categorization Quality

A stratified sample of 30 videos was selected for manual assessment across all search terms. Eligible videos were in English or Spanish, contained at least one comment, and had a duration of less than 5 minutes. The sample was stratified by search term to ensure representation across the different retrieval groups. No magnetotherapy videos met the eligibility criteria and were therefore not included in the validation sample. Video duration and number of comments were retrieved through the YouTube Data API, and video language was determined using the language-detection model described above.

Two independent annotators, both proficient in English and Spanish and with professional backgrounds in health-related fields, classified the selected videos. Interrater agreement was assessed using Cohen κ. Confusion matrices were examined to characterize the nature and direction of disagreements between the model and the human reference classification.

In addition, we conducted a targeted manual validation focused on the “cancer treatment” and “cardiac rehabilitation” search terms. Among the terms used to construct the scientific corpus, these 2 were comparatively broad, referring to general treatment or care domains rather than to a single specific intervention, and could therefore retrieve a more heterogeneous set of videos, including potentially pseudoscientific or irrelevant content that could ultimately be retained in the scientific corpus. The primary aim of this targeted assessment was to evaluate whether the use of these broad retrieval terms could result in videos that were not scientific being retained in the scientific corpus. To assess this, we examined whether videos classified as scientific by the LLM and retained by the pipeline were instead classified as pseudoscientific or irrelevant by human annotators. For this purpose, a stratified random sample of 30 English and Spanish language videos was selected, with stratification based on video language and model-assigned label. The sampled videos were independently assessed by 2 bilingual human annotators with professional backgrounds in health-related fields, blinded to the model classifications, using the same procedure as in the overall validation.

### Comment Validation Sample and Annotation Procedure

A stratified sample of 100 comments was selected for manual annotation, comprising 50 comments from each audience. Sampling was stratified by language, thematic category, and emotion. Because several thematic categories occurred only rarely in the complete dataset, not all categories were represented in the validation sample. To facilitate multilingual assessment, each comment was presented to the annotators in its original language, together with English and Spanish translations generated through the Google Gemini API using a predefined translation prompt (Multimedia Appendix 1).

### Emotion Classification

Interrater agreement for emotion classification was assessed using Cohen κ. The emotion assigned by the model was then compared separately with the classification of each annotator and with the consensus human classification using accuracy and *F*_1_-scores.

### Thematic Classification

The same 100 comments were independently reviewed by both annotators. Because comments could plausibly express more than one communicative function, annotators were allowed to select all thematic categories that they considered applicable.

Agreement between the model-assigned category and the human annotations was evaluated using several complementary measures. We calculated the proportion of comments for which the model-assigned category was selected by both annotators, by each annotator individually, and by at least one annotator.

We also calculated a weighted plausibility score ranging from 0 to 1. For each comment, each annotator contributed 0.5 points when the model-assigned category was included in their set of selected categories. Consequently, a comment received a score of 0 when neither annotator selected the model-assigned category, 0.5 when it was selected by one annotator, and 1 when it was selected by both. The mean score across comments was used as a descriptive measure of the degree of human support for the model-assigned categories, rather than as a standard inter-rater reliability coefficient.

In addition, model-annotator correspondence was evaluated separately for each annotator and for the combined human annotations by binarizing whether the model-assigned category was present in the relevant human annotation set. These measures were reported both globally and by model-assigned category.

### Qualitative Analysis of Systematic Disagreement

Following the quantitative evaluation, 4 categories were examined qualitatively. “insult” and “desires” showed no direct correspondence with either annotator, and almost all comments assigned to these categories in the annotation sample were emojis. We therefore manually inspected all comments assigned to “insult” or “desires” in the complete dataset. Most model-human no-overlap cases were concentrated in expression of feelings and comparison, whereas the remaining categories contained too few disagreement cases to support meaningful pattern identification. We therefore examined the alternative labels selected by the annotators for no-overlap cases in these 2 categories to better understand the nature of these disagreements and to identify recurrent communicative functions that may have been captured by these broad model labels. This analysis provided an empirical basis for generating more specific hypotheses about their possible meaning in the conversational patterns; however, these interpretations were treated as exploratory hypotheses.

### Final Feature Creation

For the process mining analysis, a composite feature, referred to as an “activity,” was created by concatenating the thematic category and the sentiment of each comment (eg, “thanking - positive” and “advice request - negative”). This composite feature provides a richer, more granular view of the events within a conversational flow. This approach was applied exclusively to the process mining analysis, as the use of composite features in the network analysis resulted in highly complex networks that were difficult to interpret and visualize.

Therefore, for the network analysis, a hybrid node definition strategy was adopted: only 2 categories, “expression of feelings” and “comparison,” were split by sentiment (eg, “expression of feelings - positive” vs “negative”). Together, these categories accounted for more comments than all remaining categories combined, and could have dominated the network structure if not disaggregated. This warranted their granular treatment to capture valence-specific dynamics.

### Data Analysis

#### Process Mining Analysis

The process mining was conducted using the pm4py library (version 2.7.15; Process Intelligence Solutions) in Python [48]. In this context, the collection of all conversation threads from an audience (eg, all scientific comment threads) constituted an “event log,” each individual conversation thread was treated as a “case,” and each comment within that thread, represented by its composite “activity” feature, was an “event” in the case sequence [49].

The Heuristic Miner algorithm was selected for process discovery. This algorithm is particularly well-suited for analyzing noisy and less-structured data (such as social media conversations), as it is designed to focus on the most frequent pathways while filtering out infrequent, exceptional behavior [50].

To balance model representativeness with visual interpretability, the analysis focused on a subset of the most common process variants. The number of variants included was determined by manually calibrating the complexity of the output models. We selected the maximum number of variants that maintained a comprehensible visual structure (17 for both models). This pragmatic approach ensures that the models are detailed enough to be insightful but simple enough to be interpretable.

Given the exploratory and descriptive nature of this study, process mining was applied to identify the most recurrent and structurally meaningful conversational pathways emerging from highly noisy, user-generated social media data, rather than to maximize predictive accuracy or exhaustive trace replay.

Model performance was evaluated using 2 standard fitness metrics: average fitness and percentage of fit traces [49], which were calculated considering the 500 most common variants as a prediction set. This cutoff was chosen to standardize evaluation across audiences and to focus on recurrent conversational patterns, as the remaining variants form a long tail with very low frequencies (typically occurring only once, with a minor subset in the pseudoscientific audience occurring twice). Percentage of fit traces measures the proportion of cases in the event log that can be perfectly replayed by the process model from beginning to end. Average fitness, a more nuanced metric based on token-based replay, quantifies how well the model accommodates each trace by calculating the ratio of consumed and produced tokens to missing and remaining tokens, averaged across all traces. An average fitness of 1 indicates a perfect fit. In this context, fitness metrics are interpreted as descriptive indicators of how well the discovered models represent dominant interactional structures, and moderate fitness values are therefore expected.

#### Thematic Network Analysis

As a complementary approach, thematic transition networks were constructed and analyzed using the NetworkX library (version 3.2.1; NetworkX Developers) in Python [51]. For each audience, a directed graph was created where the nodes represented the thematic categories of the comments. A directed, weighted edge was drawn from node A to node B if a comment of theme B immediately followed a comment of theme A within a conversation thread. The weight of the edge corresponds to the total number of times this specific transition occurred across the entire corpus.

To provide a quantitative comparison between the scientific and pseudoscientific conversational networks, we used the node-level metrics degree and strength. Degree represents the number of distinct connections associated with a node, whereas strength represents the sum of the weights of those connections [52]. Since the networks are directed and weighted, both metrics were calculated separately for incoming and outgoing connections, resulting in in-degree, out-degree, in-strength, and out-strength. We selected these metrics because they characterize the diversity and volume of transitions entering and leaving each thematic category. These local metrics can be interpreted directly from the observed immediate transitions. We did not consider path-based centrality measures since their interpretation relies on assumptions about how flow occurs through a network [53], whereas our aggregated networks represent immediate transitions between consecutive comments rather than complete or necessarily traversable conversational paths.

To produce interpretable network visualizations and support the qualitative comparison of the most recurrent interaction structures, an edge-weight filtering method was applied. Following the approach of Gallos et al [54], new subgraphs were generated by retaining only edges with a weight above a selected threshold. We tested multiple values for the threshold ranging from 50 to 150, which were evaluated in a sensitivity analysis, and we set this threshold at 100 transitions. This threshold represents a heuristic compromise between retaining structural transition patterns and maintaining visual interpretability in both networks. The same absolute threshold was applied to both networks to ensure that the displayed transitions met the same minimum frequency criterion.

#### Sensitivity Analyses and Parameter Calibration

To assess the sensitivity of the network visualizations to the edge-weight threshold, we repeated the filtering procedure using thresholds of 50, 75, 100, 125, and 150 transitions. For each threshold and network, we calculated the number and percentage of retained edges, the percentage of total edge weight retained, and the 5 nodes with the highest weighted strength. The same absolute threshold was evaluated in both networks to ensure that displayed transitions met a comparable minimum frequency criterion.

To assess the effect of the number of retained process variants, we calculated the cumulative proportion of conversation traces covered by the top-k most frequent variants for values of k ranging from 1 to 50. The resulting coverage curves were examined to evaluate the trade-off between additional conversation coverage and increasing visual complexity, with particular attention to diminishing incremental gains as additional variants were retained.

To assess whether the observed network patterns were driven by specific search terms, we conducted a leave-one-search-term-out sensitivity analysis. For each audience, we iteratively removed one search term and reconstructed the network. Edge weights were expressed as conditional probabilities, calculated as the number of transitions from a given source node to a specific target node divided by the total number of transitions originating from that source node. We then calculated the absolute change in each transition probability relative to the full network and summarized these differences across edges and excluded terms to evaluate pattern stability.

### Ethical Considerations

This study was conducted using publicly available data from the YouTube platform. During the data collection process, all user-identifying information was anonymized; no usernames or user IDs were stored. The final analysis and reporting are based on aggregated patterns of language and interaction, without reference to any individual users, thus ensuring user privacy. This approach aligns with ethical guidelines for research involving publicly accessible online data.

The overall workflow for comment processing and analysis is summarized in Figure 2. Comments and replies from eligible YouTube videos were collected, reconstructed into conversation threads, and processed for language, sentiment, thematic classification, process mining, and network analysis.

**Figure 2.**
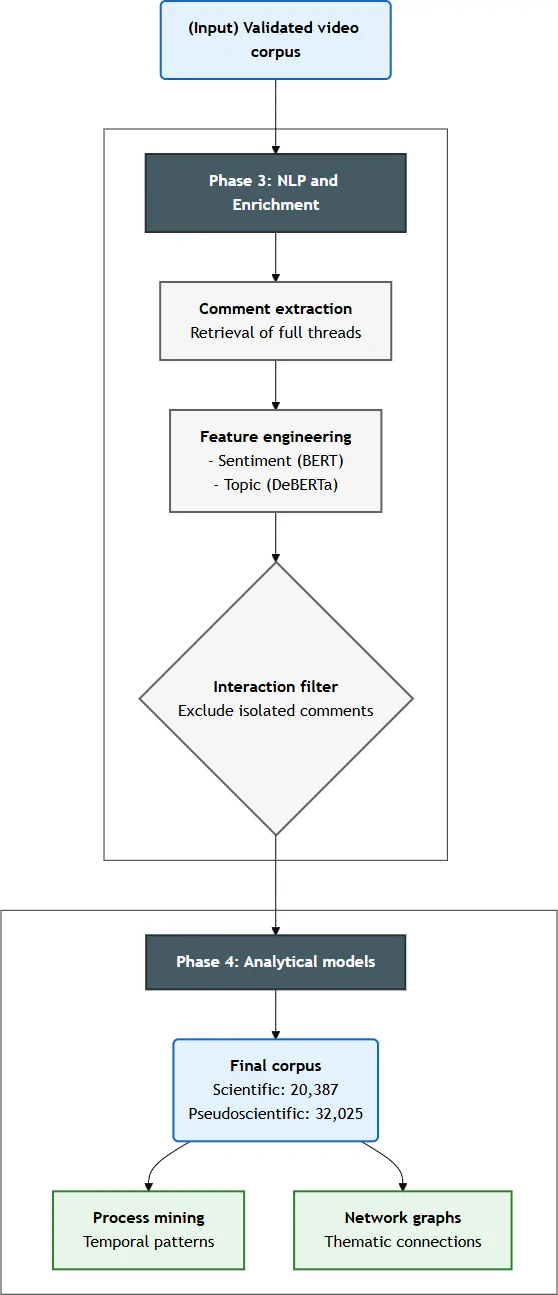
Workflow representation for obtaining the comments and analyzing them. NLP: natural language processing.

## Results

### Corpus Characteristics

The automated classification pipeline successfully categorized the collected videos, forming the basis for the 2 distinct comment corpora. The final dataset comprised 20,387 comments from videos classified as scientific and 32,025 comments from videos classified as pseudoscientific. An analysis of the temporal distribution of these comments reveals that while the data span over a decade (from 2011 to May 2025), most interactions in both corpora are concentrated in recent years, reflecting the growing use of the platform for health-related discussions. The number of comments posted each year in the scientific and pseudoscientific corpora is shown in Figure 3, where the bars represent the number of comments posted each year.

The thematic analysis revealed both structural similarities and distinct functional divergences between the two audiences. In both groups, expression of feelings and comparison were the most frequent categories. However, their relative prominence and valence differed between the two corpora. The pseudoscientific corpus showed higher frequencies of compliments, thanking, and emoji-only or brief acknowledgments, whereas medical treatment and advice requests were relatively more frequent in the scientific corpus. A detailed breakdown of category frequencies is presented in Multimedia Appendix 4.

Regarding the language of the comments, the corpora were dominated by English and Spanish. Scientific videos yielded 11,963 comments in Spanish and 4561 in English, whereas pseudoscientific videos produced 16,991 comments in English and 4645 in Spanish. Other languages accounted for 3859 comments in the scientific corpus and 10,384 in the pseudoscientific corpus. A negligible number of comments were unclassifiable. Archived live streams represented 28 of 226 (12.4%) videos and 1498 of 20,387 (7.35%) comments in the scientific corpus, compared with 56 of 461 (12.1%) videos and 5942 of 32,025 (18.55%) comments in the pseudoscientific corpus.

**Figure 3.**
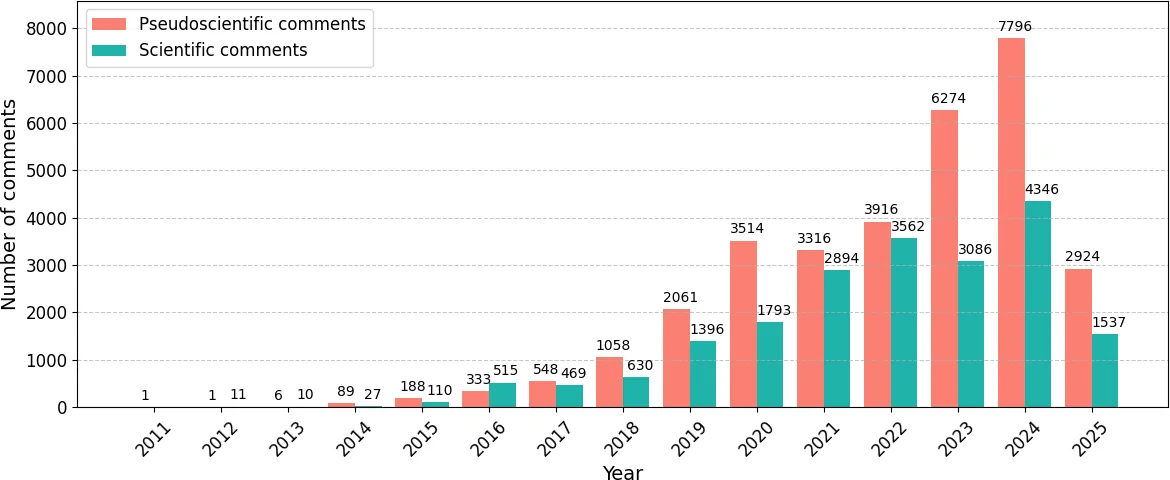
Histogram of comments per year in the scientific and pseudoscientific audiences.

### Quality of the Video and Comment Classifications

#### Video Classification

Cohen κ between both annotators was 0.83. The accuracy and the *F*_1_-score of the first annotator and the LLM were 0.86 and 0.72, respectively, and for the second annotator, 0.76 and 0.64. Table 2 compares the classifications assigned by the model and the 2 annotators.

Rows represent the categories assigned by the LLM, while columns represent the categories assigned by each annotator. Most disagreements concerned the application of inclusion and exclusion criteria rather than confusion between the scientific and pseudoscientific categories. Three videos classified as irrelevant by the LLM were classified as scientific by both annotators, and 3 videos classified as pseudoscientific by the LLM were classified as irrelevant by annotator 2. Only one direct disagreement between the scientific and pseudoscientific categories was observed: annotator 2 classified one video as pseudoscientific that the LLM had classified as scientific.

The targeted manual validation of the “cancer treatment” and “cardiac rehabilitation” search terms showed that 21 of the 30 sampled videos had been classified as scientific by the LLM, and all 21 were also classified as scientific by both human annotators. Disagreements were observed only among videos that the LLM had classified as pseudoscientific or irrelevant. Under the filtering procedure used in this study, these videos were excluded and were therefore not retained in the final corpus. Consequently, the only sampled videos retained by the pipeline were the 21 videos for which the LLM and both human annotators agreed on the scientific classification. These findings provide no evidence that the use of these broader scientific search terms resulted in pseudoscientific or irrelevant videos being retained in the scientific corpus.

**Table 2.** Comparison of LLM^a^ video classification with the classifications of 2 human annotators.

LLM category	Annotator 1			Annotator 2		
	Scientific	Pseudoscientific	Irrelevant	Scientific	Pseudoscientific	Irrelevant
Scientific	11	0	0	10	1	0
Pseudoscientific	0	14	1	0	12	3
Irrelevant	3	0	1	3	0	1

a LLM: large language model.

#### Emotion Classification of the Comments

Cohen κ between both annotators was 0.75, and the accuracy and the *F*_1_-score for the first annotator were 0.68 and 0.6, respectively, and for the second annotator were 0.6 and 0.52. The corresponding confusion matrices are presented in Table 3.

The confusion matrices showed moderate correspondence between the model and both annotators. Agreement was highest for positive comments, whereas negative and neutral comments were more frequently confused with one another. This pattern was similar for both annotators, although annotator 2 classified a larger proportion of model-negative comments as neutral. Overall, the results suggest that the model distinguished positive emotion more consistently than the boundary between negative and neutral emotion.

**Table 3.** Confusion matrices comparing model-based emotion classification with 2 human annotators.

Model category	Annotator 1			Annotator 2		
	Positive	Negative	Neutral	Positive	Negative	Neutral
Positive	44	5	3	41	4	7
Negative	6	16	13	5	12	18
Neutral	2	2	7	3	2	6

### Thematic Categories Plausibility

Of the 100 comments initially sampled, 2 were excluded because they contained only user mentions and no substantive text for thematic assessment. Among the remaining 98 comments, the model-assigned category was selected by annotator 1 in 48 (49%) cases and by annotator 2 in 35 (35.7%) cases. At least one annotator selected the model-assigned category in 52 (53.1%) cases, whereas both annotators selected it in 31 (31.6%) cases.

Because the objective was to assess the plausibility of the model-assigned categories in a multilabel setting, rather than requiring complete agreement between both annotators, we additionally calculated a score for estimating the plausibility of the tags that assigned partial credit when the category was selected by only one annotator and full credit when it was selected by both.

For each comment, the score for estimating the plausibility was calculated as the proportion of annotators who included the model-assigned category in their set of selected labels. Each annotator contributed 0.5 points: the score was 0 when neither annotator selected the category, 0.5 when it was selected by one annotator, and 1 when it was selected by both. The overall plausibility score was obtained by averaging the comment-level scores across the 98 comments. This measure was used as a descriptive indicator of human support for the plausibility of the model-assigned category. The resulting overall score was 0.423.

Table 4 presents category-specific model-annotator correspondence, including the estimated plausibility score and the proportion of comments for which the model-assigned category matched the human annotations.

Table 4 shows model-annotator correspondence for each thematic category. Performance varied substantially across labels. “thanking” and “greetings” received complete support from both annotators, while “medical treatment” also showed high correspondence. Intermediate support was observed for “expression of feelings*”* and “compliment.” In contrast, “comparison,” “speculation,” and “advice request*”* showed limited correspondence, particularly for annotator 2, whereas “desires*”* and “insult*”* received no direct support from either annotator. The estimated plausibility score summarizes the proportion of annotators who selected the model-assigned category, assigning partial credit when only one annotator selected it.

**Table 4.** Category-specific human support for zero-shot classifier–assigned thematic labels.

Model-assigned category	n	Selections annotator 1	Selection percentage annotator 1	Selections annotator 2	Selection percentage annotator 2	Weighted plausibility percentage
Thanking	7	7	100	7	100	100
Greetings	2	2	100	2	100	100
Medical treatment	3	2	66.7	3	100	83.33
Advice given	1	1	100	0	0	50
Expression of feelings	40	20	50	16	40	45
Compliment	10	4	40	5	50	45
Comparison	23	10	43.5	2	8.7	26.1
Speculation	2	1	50	0	0	25
Advice request	3	1	33.3	0	0	16.7
Desires	4	0	0	0	0	0
Insult	3	0	0	0	0	0

### Analysis of Classifier-Human Disagreement

The inspection of comments assigned to “insult” and “desires” showed that these categories predominantly captured emoji-only comments, especially hearts, praying hands, and smiling faces, together with brief acknowledgments or very short reactions. Based on this pattern, both categories were merged for the subsequent exploratory analysis as emoji-only or brief acknowledgment.

Most model-human no-overlap cases were concentrated in expression of feelings and comparison. A detailed analysis of these disagreements, including human agreement within no-overlap cases and the distribution of alternative labels jointly selected by both annotators, is provided in Multimedia Appendix 5. Shared human labels were relatively diffusely distributed across alternative categories for expression of feelings. In contrast, for comparison, 75% (15/20) of the human labels jointly selected by both annotators in no-overlap comments corresponded to advice given, criticism, medical treatment, or speculation.

### Process Models of Audience Interaction

The application of process mining revealed starkly different conversational “blueprints” for the scientific and pseudoscientific audiences. These models visualize the most frequent sequences of interactions, with arrows indicating the flow of conversation and numbers representing the frequency of each transition.

The cumulative-coverage analysis showed progressively smaller gains as additional process variants were included. At k=17, the retained variants covered approximately 36% (4129/11,338) of conversations in the pseudoscientific corpus and 22% (1281/5800) in the scientific corpus. Beyond this point, the incremental increase in coverage became smaller, while additional variants increased the complexity of the process models. Therefore, 17 variants were retained for both audiences.

The quantitative results of the analysis were as follows: the scientific corpus contained 5800 conversation threads with 2505 distinct variants (variant-to-case ratio=0.43), while the pseudoscientific corpus contained 11,338 threads with 3019 variants (ratio=0.27), indicating greater conversational diversity in scientific discussions. Variant frequencies followed a long-tail distribution: 457 scientific variants (accounting for 64.69% of conversations) and 697 pseudoscientific variants (accounting for 79.52% of conversations) occurred more than once, with the majority of the variants appearing only once.

In terms of fitness, the process model trained on the scientific-audience comments achieved an average fitness of 0.79 and a percentage of fit traces of 42.53%, indicating that 42.53% (1614/3795) of the observed conversation traces conform to the model. The model trained on the pseudoscientific audience comments obtained an average fitness of 0.81 and a percentage of fit traces of 42.09% (3629/8622). Although these values are moderate, they are consistent with our exploratory goal: in noisy, user-generated social media data, the discovered models are intended to capture general descriptive behavioral patterns rather than enable deterministic predictions. A summary of the main characteristics of both process models is provided in Table S1 in Multimedia Appendix 6.

### Scientific Audience

Figure 4 shows the process model for the scientific audience. Nodes represent conversational activities, defined by thematic category and sentiment, while directed arrows indicate transitions between consecutive activities within a conversation, and numbers indicate transition frequencies. The green and orange nodes represent the start and end of a conversation trace, respectively. The model displays the 17 most frequent process variants. Initial transitions are concentrated in negative expressions of feelings (n=465), positive compliments (n=305), positive expressions of feelings (n=290), and negative comparisons (n=221). Negative states show greater persistence and diversification, whereas positive states tend to lead to thanking, brief acknowledgments, or closure. The final node receives transitions from nearly all categories.

**Figure 4.**
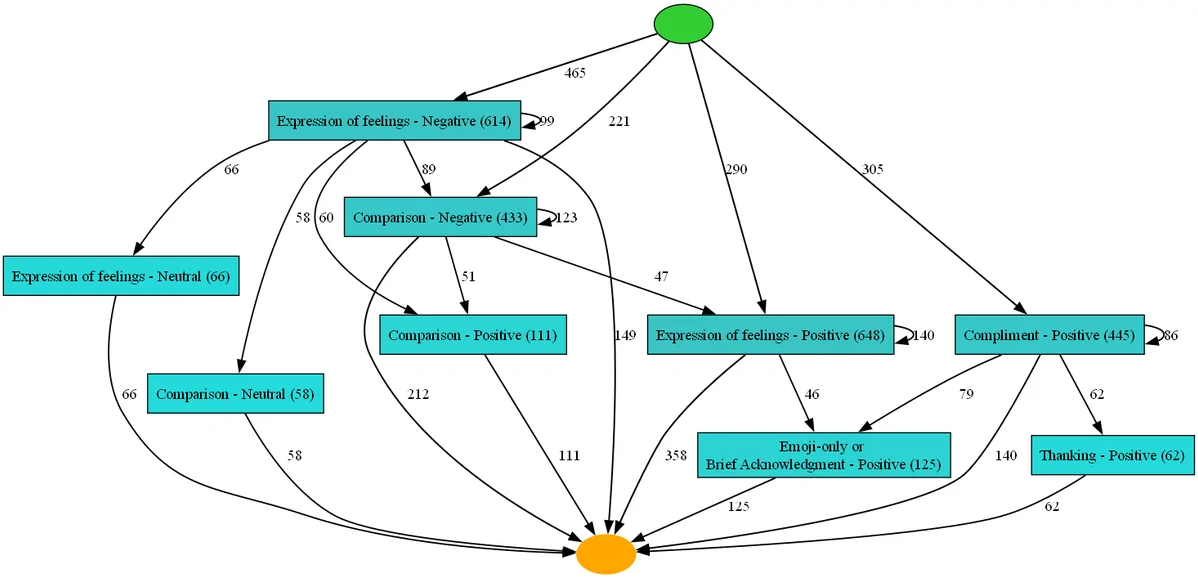
Process detected for the scientific audience.

### Pseudoscientific Audience

Figure 5 shows the process model for the pseudoscientific audience, using the same representation conventions described for Figure 4. Initial transitions are concentrated in positive expressions of feelings (n=1838), thanking (n=929), positive compliments (n=868), and emoji-only responses or brief positive acknowledgments (n=183). Positive expressions of feelings display the highest self-transition frequency (n=630) and also lead frequently to the final state (n=1121). The largest direct transition to the final state originates from emoji-only responses or brief positive acknowledgments (n=1207), followed by positive expressions of feelings (n=1121) and positive compliments (n=689).

**Figure 5.**
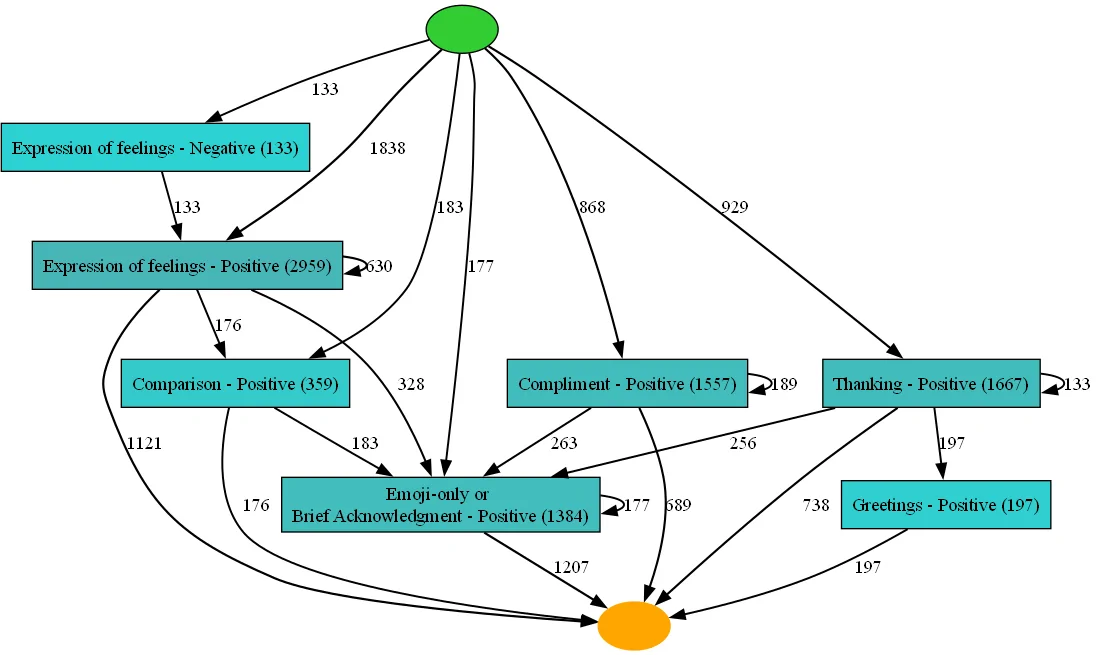
Process detected for the pseudoscientific audience.

### Thematic Transition Networks

Complementary to the process-mining results, the network analysis summarized the frequency and direction of immediate transitions between thematic categories. Regarding the node-level metrics, in-degree and out-degree were at or near their maximum values for most nodes in both networks, indicating that most thematic categories were connected to nearly all other categories at least once. The comparison therefore focused on normalized in-strength, out-strength, and total strength, which capture the relative volume of transitions involving each category. Detailed node-strength metrics for both networks are provided in Table S2 in Multimedia Appendix 6.

The threshold sensitivity analysis showed that the 5 nodes with the highest weighted strength remained unchanged across all examined thresholds in both networks. Lower thresholds retained more edges and total weight but produced denser visualizations, whereas higher thresholds progressively removed a substantial proportion of the total transition weight. At the selected threshold of 100, the pseudoscientific network retained 49 edges, representing 8.4% (49/583) of all possible observed edges and 66.49% (13,755/20,687) of the total edge weight, while the scientific network retained 37 edges, representing 6.92% (37/535) of edges and 54.37% (7931/14,586) of total weight. These results supported the use of 100 transitions as a pragmatic balance between preserving the main weighted structure and maintaining visual interpretability. Detailed results are provided in Table S1 in Multimedia Appendix 7.

### Visual Comparison of the Filtered Networks

The analysis of edge weight distribution (Multimedia Appendix 8) of the initial network that was applied after creating it showed that both networks were dominated by many very weak ties (transitions that occurred only a few times), with a small number of very strong ties accounting for the bulk of the interactions. By filtering out edges with a weight of less than 100, we were able to isolate the core conversational backbone of each community.

### Scientific Audience Network

The network obtained for this audience is displayed in Figure 6. Nodes represent thematic categories, and directed edges represent immediate transitions between consecutive comments. Edge weights correspond to the frequency of each transition. Only transitions occurring at least 100 times are shown. Three nodes were most prominent: expression of feelings - negative, expression of feelings - positive, and comparison - negative. These nodes showed multiple incoming and outgoing connections, strong mutual connectivity, and visible self-loops. Compliment and comparison - positive occupied intermediate positions and were connected to several of the dominant nodes. The remaining categories, including expression of feelings - neutral, comparison - neutral, medical treatment, advice request, and emoji-only or brief acknowledgment, were more peripheral and displayed fewer or weaker connections.

**Figure 6.**
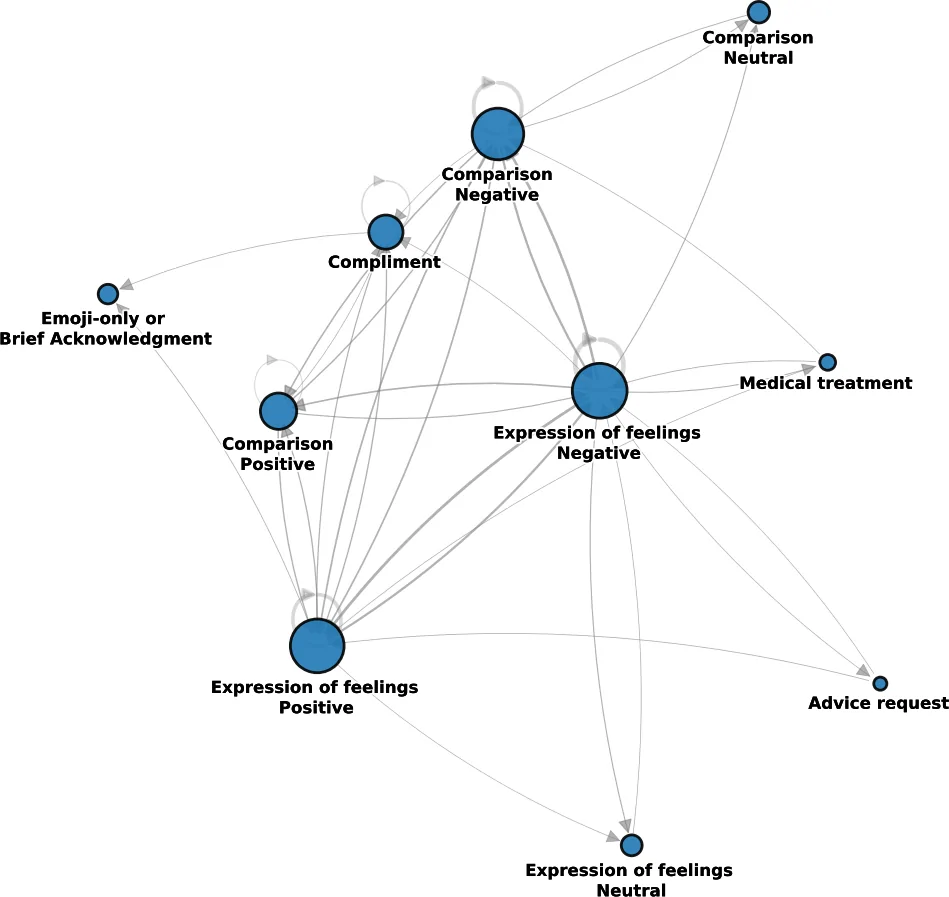
Network of the scientific audience interactions.

### Pseudoscientific Audience Network

The network obtained for this audience is displayed in Figure 7, using the same representation conventions described for Figure 6. “expression of feelings - positive” leads by size and global connectivity. “expression of feelings - positive” was the most prominent node and showed extensive incoming and outgoing connectivity, including a visible self-loop. Emoji-only or brief acknowledgment, compliment, thanking, and comparison - positive also occupied prominent positions and were densely connected with one another and with positive expressions of feelings. “comparison - negative” and “expression of feelings - negative” were somewhat less prominent but remained integrated into the main network structure. “comparison - neutral,” “expression of feelings - neutral,” and greetings were more peripheral and showed fewer or weaker connections.

**Figure 7.**
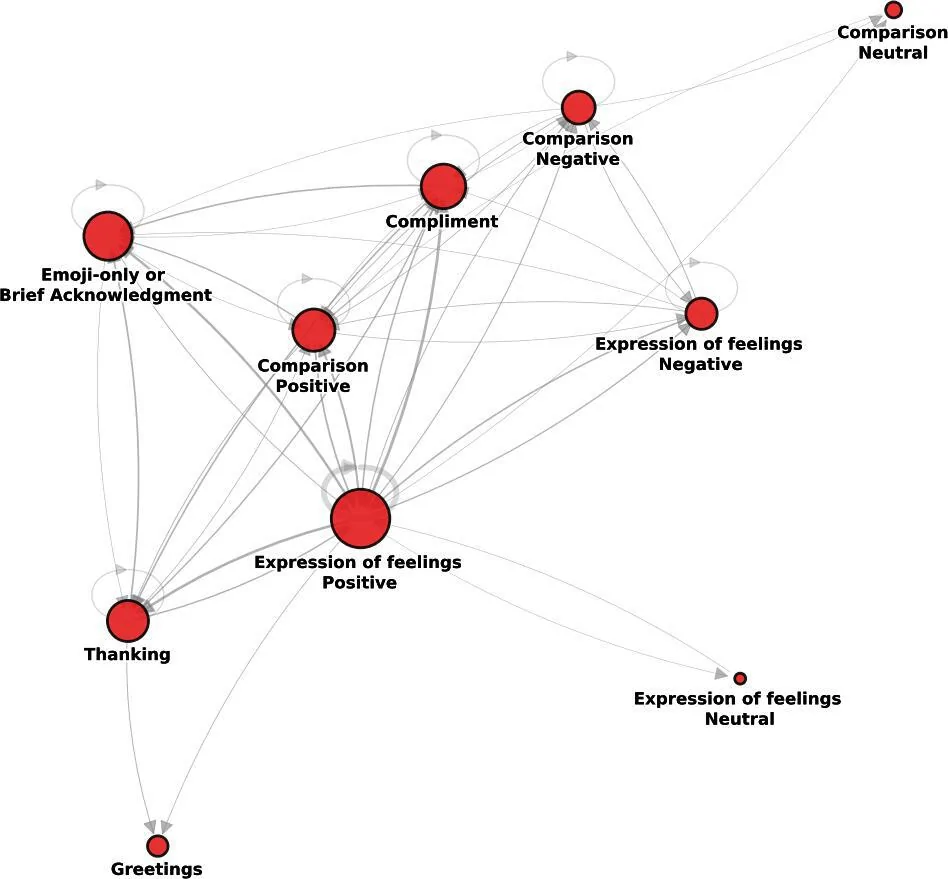
Network of the pseudoscientific audience interactions.

### Results of the Sensitivity Analysis of the Patterns Found in the Networks

The leave-one-search-term-out sensitivity analysis showed that most transition probabilities were stable after excluding individual search terms. Median absolute changes remained below approximately 1‐2 percentage points across terms, and most transitions changed by less than 5 percentage points. Larger deviations were concentrated in a limited number of transitions, particularly after excluding Reiki and cancer treatment, and to a lesser extent acupuncture and cognitive behavioral therapy. These findings suggest that no single search term broadly determined the structure of either network, although some local transitions were sensitive to the exclusion of specific topics.

## Discussion

### Principal Results

This study used process mining and network analysis to identify and compare conversational patterns across 2 distinct corpora of scientific and pseudoscientific health discussions on YouTube, moving beyond content analysis to examine sequential and structural features of conversations. In the following sections, we discuss several of the principal patterns identified in the 2 retrieved corpora. These findings should not be interpreted as an exhaustive characterization of scientific or pseudoscientific health discourse, but as corpus-specific patterns that illustrate how the proposed methodology can detect meaningful differences between discussions surrounding health topics with contrasting scientific framing.

### Pseudoscientific Audience

In the pseudoscientific corpus examined in this study, the process model revealed a pattern dominated by 4 positive activities, “expression of feelings,” “thanking,” “compliment,” and “emoji-only or brief acknowledgment,” with “expression of feelings - negative” as the only negative activity represented in the model. The network analysis showed a similar configuration: the largest nodes corresponded to the principal activities identified in the process model, although negative comparison and negative expression of feelings also showed relatively high strength. Together, the models depict generally positive interactions while also retaining some visible negative interactions.

One possible interpretation of this predominance of positive activities is that the interactions may reflect an interaction style centered on mutual validation and socioemotional bonding, in which users frequently disclose feelings and receive positive social feedback through gratitude and compliments.

Previous research provides some support for this interpretation. Gratitude expressions can function as relational signals that facilitate social affiliation via perceived warmth, which could support the interpretation of “thanking” as a possible bond-maintenance mechanism [55]. The prominence of “expression of feelings - positive” may include cases of emotional self-disclosure, that is, users sharing personal experiences and the thoughts and feelings attached to those experiences [56]. Prior research on self-disclosure in social networking contexts suggests that greater self-disclosure is associated with higher perceived familiarity and interpersonal closeness with others in the social environment [56]. However, this interpretation should be treated cautiously because the category is broad and may encompass a wide range of affective statements rather than a single, narrowly defined communicative act. This heterogeneity was also reflected in the model-annotator disagreement analysis: among no-overlap cases, the alternative labels jointly selected by both annotators were relatively diffusely distributed, without a clear concentration in a particular set of labels. Taken together, this pattern is consistent with the exploratory hypothesis that the model may have used expression of feelings as a broad umbrella for comments containing subjective or affective content.

“comparison - positive” also formed part of this predominantly positive structure, but its specific communicative meaning should be interpreted cautiously because the category showed limited correspondence with the human annotations and may encompass several different functions.

One hypothesis is that the classifier used this label to capture a broader evaluative function rather than explicit comparison. This interpretation is consistent with the qualitative disagreement analysis: among the human labels jointly selected by both annotators for no-overlap cases classified by the model as comparison, 75% (15/20) corresponded to advice given, criticism, medical treatment, or speculation. These categories suggest evaluative, interpretive, or analytical functions rather than emotional ones. However, this interpretation also remains exploratory for this category.

“emoji-only or brief acknowledgment” also occupied a prominent position. In the process model, this activity frequently appeared immediately before the end of a conversation. Consistently, the network analysis showed substantially greater normalized in-strength than out-strength for this category (12.89 vs 6.19), indicating that it more frequently received transitions from other activities than generated subsequent transitions. Taken together, these findings support the hypothesis that emoji-only responses and brief acknowledgments may be associated with a lower tendency for conversational continuation and may often function as terminal or near-terminal responses. Within the corpus examined here, these responses may therefore function as simple affective signals that acknowledge preceding comments without prompting further interaction.

### Scientific Audience

The network was structured around 4 prominent nodes corresponding to positive and negative expressions of feelings and comparison. However, “comparison - negative” showed substantially greater normalized strength than “comparison - positive” (15.39 vs 8.87), while “expression of feelings - negative” was also slightly more prominent than its positive counterpart (17.51 vs 15.39). Compared with the stronger positive-valence concentration observed in the pseudoscientific corpus, the scientific network displayed a more mixed-valence structure, with a relative prominence of negative expression of feelings and negative comparison.

One possible interpretation is that conversations in the scientific sample more frequently incorporated problem-focused, critical, or evaluative content. The relative prominence of “comparison - negative” and “expression of feelings - negative,” together with the greater visibility of “medical treatment,” may reflect discussions in which users describe difficulties, assess treatments, or question outcomes. As previously discussed, the comparison category may capture a broader range of evaluative, critical, or interpretive functions. Although advice request also showed greater normalized strength in the scientific sample, it was not used as a primary basis for interpretation because it received limited human support and was represented by few cases in the annotation sample.

Notably, thanking was substantially less prominent in the scientific network than in the pseudoscientific network, as reflected in its normalized total strength (2.98 vs 9.72). Medical treatment showed the opposite pattern, with greater normalized strength in the scientific network (4.57 vs 0.98). Conversely, emoji-only or brief acknowledgment was less prominent in the scientific network (3.80 vs 9.54). Taken together, these differences are consistent with a relatively greater presence of treatment-related content in the scientific network, whereas gratitude and simple affective signals were comparatively more prominent in the pseudoscientific network.

The process model also shows several recurrent pathways involving both affective and evaluative activities. “expression of feelings - negative” was the most frequent starting activity, followed by “comparison - negative,” “expression of feelings - positive,” and “compliment - positive.” Although several pathways began with negative expressions or comparisons, subsequent transitions included positive, neutral, and negative categories, and “expression of feelings - positive” was the activity that most frequently preceded conversational closure. This pattern may reflect a form of conversational de-escalation, whereby negative content did not systematically trigger further negativity and was sometimes followed by more neutral or positive exchanges before closure. In addition, endings were more broadly distributed across activities in the scientific sample than in the pseudoscientific sample, where closure was more strongly concentrated around “emoji-only or brief acknowledgment.” This broader distribution of terminal activities suggests greater heterogeneity in how conversations concluded.

### Comparison With Prior Work

Prior studies show that exposure to misinformation within interactive peer-led communities was associated with greater adherence to the misinformation circulating in those communities [57]. The patterns identified in the pseudoscientific corpus examined in this study are compatible with processes of interpersonal reinforcement and, in some contexts, could contribute to the maintenance of echo-chamber dynamics. The predominance of positive interactions (thanking, compliments, expressions of feelings - positive, and emoji-only or brief acknowledgments) in the pseudoscientific corpus may be consistent with a conversational environment particularly reinforcing for users seeking social affirmation. Similar forms of positive social feedback have been associated with trust and interpersonal closeness in other online communities [58]. The scientific corpus showed a more mixed-valence structure and a greater relative presence of treatment-related content, which is less consistent with this hypothesis.

Furthermore, our results offer a nuanced perspective on the limitations of fact-checking highlighted by Wasike [13]. As discussed above, the interaction patterns identified in the extracted pseudoscientific corpus suggest that emotional support and social validation may play a role in these conversations. If some engagement in certain pseudoscientific communities is partly sustained by emotional support and social validation, interventions based solely on factual correction may be insufficient because they do not address these interactional dimensions. This aligns with broader findings in health communication that suggest simply debunking misinformation is not enough to change minds [8].

This study also contributes to the growing body of research that uses computational methods to analyze social media health discussions [31]. While other studies have effectively used NLP for sentiment analysis on YouTube comments [59], combining process mining with thematic network analysis adds a temporal and structural dimension. This shifts the analytical focus from a static inventory of what is said to an examination of how communicative activities are ordered and connected within conversational threads.

### Limitations

This study has several limitations. First, the findings are specific to the YouTube platform. The conversational dynamics on other social media sites, such as Twitter, Facebook, or Reddit (Reddit, Inc), may differ significantly due to variations in platform architecture, user demographics, and communication norms. Recent cross-platform research has shown that user engagement varies according to how users perceive platform characteristics, and discussions of the same topic may exhibit different thematic and engagement patterns across platforms [60,61]. More broadly, features shaping the user experience, together with differences in content format and temporal context, were not systematically measured or stratified in this study, and may have influenced user behavior and the conversational patterns identified. In addition, the study examined interactions among commenters rather than exposure, recommendation, sharing, audience retention, or longitudinal view trajectories. The identified conversational patterns should therefore not be interpreted as predictors of video reach or virality.

Second, the analysis relies on automated models for emotion and thematic classification. Human validation showed substantial inter-rater agreement for emotion classification, while model-human correspondence was generally stronger for positive comments than for the distinction between negative and neutral comments. For thematic classification, the overall plausibility score was 0.423, indicating moderate human support for the model-assigned labels. However, plausibility varied substantially across categories, with some labels showing strong correspondence with human annotations and others receiving more limited support.

The manual inspection of comments assigned to “insult” and “desires,” which showed no overlap with the human annotations, clarified that these categories mainly captured emoji-only comments and brief acknowledgments. Merging them into “emoji-only or brief acknowledgment” enabled more meaningful comparisons between the two samples than retaining the labels without interpretation. However, the category remains broad, and a more granular classification would allow a clearer understanding of the different communicative functions grouped within it. Similarly, the qualitative analysis of disagreements involving expression of feelings and comparison supported exploratory hypotheses about the functions captured by these labels, although these interpretations may not apply uniformly to all cases. These findings highlight the need for a context-specific functional taxonomy better suited to multidimensional social media conversations.

Third, the corpus was assembled through a convenience-based retrieval strategy using a limited set of predefined topics. The search strategy was not designed to identify or comprehensively represent the most prevalent or prominent scientific and pseudoscientific health topics on YouTube, and other potentially relevant treatment domains were therefore not included. This selective topical coverage limits the generalizability of the findings beyond the retrieved corpora. The leave-one-search-term-out sensitivity analysis showed that no single term broadly determined the network structures, although some local transitions were sensitive to the exclusion of specific topics. However, this analysis does not establish that the overall corpus is representative of scientific or pseudoscientific health discourse. In addition, the unequal language distribution between the samples may partly reflect language-specific patterns. The findings should therefore be interpreted as descriptive of the retrieved data and as serving the purpose of testing the proposed methodology across contrasting forms of health discourse, rather than as general characteristics of scientific and pseudoscientific discourse.

Another limitation concerns the use of single-label classification. Individual comments may simultaneously express multiple topics, emotions, and communicative functions, whereas the model assigns only one dominant category to each comment. This necessarily reduces semantic complexity and may obscure additional relevant dimensions of the interaction. As a result, the sequential analysis captures the dominant assigned category rather than the full range of meanings present in each contribution, limiting the granularity of the resulting pathways. Moreover, zero-shot classification performance can vary across tasks, classes, and languages and may depend on how candidate labels are formulated [43,44]. Consistent with this, correspondence with human annotations varied considerably across categories, indicating that some labels were captured more reliably than others. Together, these limitations reduce the granularity of the resulting conversational pathways and warrant greater caution when interpreting categories with lower human correspondence.

Beyond these study-specific constraints, the reliance on LLMs for data annotation introduces specific challenges. While LLMs provide scalability for analyzing vast datasets, they may reflect inherent training biases, potentially affecting the interpretation of culturally nuanced narratives or sarcasm [62-64]. Furthermore, the closed nature of proprietary models limits full transparency regarding their internal decision-making processes [64]. In this study, we mitigate these concerns by documenting the exact model, configuration, and prompts used, and we assess classification reliability through human validation and targeted manual inspection of model outputs. Future studies could further reduce these limitations by auditing potential biases and comparing proprietary models with open-source alternatives whose parameters can be frozen and independently examined.

Taken together, these limitations define the exploratory scope of the study. Within these methodological boundaries, the analyses identified interpretable differences between the two corpora that remained visible across the filtering and sensitivity procedures applied. These findings should therefore be interpreted as evidence that the proposed approach can detect potentially meaningful contrasts in conversational structure, rather than a generalizable characterization of the audiences. Their principal value lies in generating hypotheses and identifying patterns that can be examined in future studies using more granular classifications, broader samples, and prospective or cross-platform designs.

### Implications for Public Health and Science Communication

The findings of this study have potential implications for public health organizations, science communicators, and clinicians.

Previous research indicates that fact-checking may have limited effectiveness in reducing misinformation sharing on social media, while attempts to debunk science-relevant misinformation have, on average, also shown limited success [12-15]. This suggests that interventions focused only on correcting content may overlook the social dimension of how users interact around misinformation. In this study, the conversational patterns identified in the pseudoscientific corpus generated hypotheses about social affirmation and interpersonal reinforcement. Although exploratory, these findings illustrate how conversational analysis may identify forms of affirmation, reinforcement, or contestation within specific audiences, providing a basis for adapting communication strategies.

Second, the scientific corpus showed strong interconnections among positive and negative expressions of feelings and comparison, together with greater visibility of medical treatment, suggesting that emotional, evaluative, and treatment-related content frequently occurred within the same conversational pathways. For health communicators and clinicians, this highlights the potential value of addressing uncertainty, treatment experiences, and concerns about outcomes directly. More broadly, these findings illustrate how the proposed approach may complement social media listening by adding a sequential perspective to the analysis of public discourse [25]. Conversational analysis can additionally examine how these elements are related across interactions. Health-related decisions are shaped not only by the information individuals encounter, but also by social relationships, interpersonal advice, and social norms [65]. By examining how conversational elements unfold and relate across interactions, the proposed approach may help identify audience-specific patterns and generate hypotheses to inform more context-sensitive communication strategies.

### Future Research

Several promising directions emerge for future research building on the foundations laid by this study. First, future work should extend the analysis beyond YouTube to other social media, such as Twitter, Facebook, or Telegram (Telegram FZ-LLC). Cross-platform and longitudinal studies stratified by topic, content format, language, and time period could assess whether conversational patterns vary across digital environments and whether they are associated with measures of diffusion, including reach, sharing, audience retention, and thread growth.

Second, future research should characterize the relative prevalence of different topics and treatments in online health discourse to support more systematic and representative sampling strategies. Subsequent studies could then assess whether the conversational patterns identified here remain stable or vary across topics, treatments, and levels of online prominence.

Third, further improvements in methodological approaches are also needed. The use of a single heuristic miner algorithm for process mining could be complemented or replaced by testing other algorithms such as inductive [66] or fuzzy miners [67]. Richer network analysis approaches could also be examined, including bipartite networks connecting comment emotions or categories to users. Such analyses could help determine whether particular conversational patterns are broadly distributed across participants or concentrated among specific users or interaction structures [68].

Fourth, future research should refine the taxonomy used to classify comments and conversational behaviors. This could include distinguishing emoji-only comments from brief acknowledgments, developing more granular categories to reduce the overuse of broad labels, and exploring data-driven category development followed by expert validation. Multilabel approaches could also better capture the multidimensional nature of comments. Multiperspective trace-encoding methods, such as those described by Rullo et al [69], could further integrate sequential, comment-level, and thread-level information to support richer process mining and network analyses.

Future research could also combine the computational pipeline with systematic qualitative analysis. After relevant conversational patterns are identified, selected patterns could be examined by domain experts. This could help distinguish algorithmically detected structures from their substantive meaning and support more valid interpretations of conversational behavior.

### Conclusions

This study applied process mining and network analysis to compare conversational patterns across 2 distinct corpora of scientific and pseudoscientific health discussions on YouTube. The two corpora differed in the relative prominence and sequencing of thematic and emotional categories. Within the corpora examined, scientific discussions showed a broader mixture of positive and negative expressions, comparisons, and treatment-related content, whereas pseudoscientific discussions showed greater prominence of positive expressions, thanking, and emoji-only or brief acknowledgment responses. Beyond these empirical differences, one possible interpretation is that the former may reflect more evaluative or problem-focused exchanges, whereas the latter may be consistent with greater interpersonal affirmation or socioemotional bonding. These interpretations should be understood as hypotheses generated from the observed patterns rather than as direct evidence of underlying psychological mechanisms. Sequential analysis may therefore provide a useful basis for examining how health-related claims and concerns are reinforced, challenged, or developed through interaction.

Future studies should test whether these patterns replicate across topics, platforms, and populations and whether they can inform context-sensitive communication strategies.

## Supplementary material

10.2196/95273 Multimedia Appendix 1 Prompts used by the large language models.

10.2196/95273 Multimedia Appendix 2 Search terms, video counts, and comment volumes by treatment category.

10.2196/95273 Multimedia Appendix 3 Categories used for zero-shot classification.

10.2196/95273 Multimedia Appendix 4 Breakdown of the category frequencies of the comments by audience.

10.2196/95273 Multimedia Appendix 5 Detailed analysis of classifier-human disagreement in thematic classification.

10.2196/95273 Multimedia Appendix 6 Process mining summary and thematic network metrics for scientific and pseudoscientific discussions.

10.2196/95273 Multimedia Appendix 7 Sensitivity analyses of network thresholds, retained process variants, and search-term effects.

10.2196/95273 Multimedia Appendix 8 Probability of weight links for the network of the scientific and the pseudoscientific audience.

10.2196/95273 Checklist 1 Completed STROBE checklist.

## Acknowledgments

CRISP-DM: Cross-Industry Standard Process for Data Mining
IMRD: Introduction, Methods, Results, and Discussion
LLM: large language model
NLP: natural language processing
SIP: sequential information processing
STROBE: Strengthening the Reporting of Observational Studies in Epidemiology
