# Supplementary material for: Exploring Conversational Dynamics in Scientific and Pseudoscientific Health Communities on YouTube: Process Mining and Network Analysis Study
Source: J Med Internet Res. 2026 Oct 2;28:e95273. doi: 10.2196/95273 (PMC13633066; doi:10.2196/95273)
Supplement: Supplementary file 1 [file jmir-v28-e95273-s001.docx]

**LLM Prompt for Video Classification**

The following verbatim prompt was provided to the Google Gemini model (gemini-1.5-flash-latest) to classify each video:

| *Original language* | *English Translation (for Clarity)* |
| --- | --- |
| *A continuación, recibirás la información básica de un vídeo de YouTube que deberás usar para clasificar el vídeo en una de las siguientes categorías:*  *Científico: El vídeo presenta información basada en evidencia empírica, métodos y consenso científicos.*  *Pseudocientífico: El vídeo presenta información que se disfraza de ciencia, pero carece de evidencia empírica, métodos científicos rigurosos o consenso científico.*  *Irrelevante: El contenido del vídeo no está relacionado con ciencia o pseudociencia, o es difícil de clasificar debido a su ambigüedad o falta de claridad.*  *Si la información provista es muy breve y genérica, clasifícalo como irrelevante. Devuelve únicamente la etiqueta de la categoría. Escribe exclusivamente la palabra, no quiero que haya nada más tras ella. Ni siquiera un salto de línea o un punto.*  *A continuación, se provee el título del vídeo, la descripción del mismo, y sus etiquetas. En ocasiones alguno de estos campos puede estar vacío. En este caso haz la evaluación con la información que tienes disponible. Si no hay información suficiente para determinar con precisión la categoría, simplemente clasifícalo como "Irrelevante".*  *Título: {title}*  *Descripción del vídeo: {description}*  *Etiquetas del vídeo: {tags}* | *You will receive basic information about a YouTube video that you must use to classify the video into one of the following categories:*  *Scientific: The video presents information based on empirical evidence, scientific methods, and consensus.*  *Pseudo-scientific: The video presents information that is disguised as science but lacks empirical evidence, rigorous scientific methods, or scientific consensus.*  *Irrelevant: The video's content is not related to science or pseudoscience, or it is difficult to classify due to ambiguity or lack of clarity.*  *If the information provided is very brief and generic, classify it as irrelevant. Return only the category label. Write exclusively the word; I do not want anything else after it. Not even a line break or a period.*  *Below is the video title, its description, and its tags. Sometimes one of these fields may be empty. In that case, perform the evaluation with the information you have available. If there is not enough information to accurately determine the category, simply classify it as "Irrelevant".*  *Title: {title}*  *Video Description: {description}*  *Video Tags: {tags}* |

**Translation prompt used for multilingual comment annotation**
To support the manual annotation of multilingual comments, each comment was translated into English and Spanish using the following prompt, with the target language specified as appropriate:

You are a professional translator.

Translate the following text into {target_language}.

Preserve the original meaning and tone. Do not summarize, explain, or add information.

Return only the translated text.

Text:
{comment}
