# Supplementary material for: Exploring Conversational Dynamics in Scientific and Pseudoscientific Health Communities on YouTube: Process Mining and Network Analysis Study
Source: J Med Internet Res. 2026 Oct 2;28:e95273. doi: 10.2196/95273 (PMC13633066; doi:10.2196/95273)
Supplement: Supplementary file 2 [file jmir-v28-e95273-s002.docx]

The table below summarizes the number of videos and comments extracted from each pseudo-scientific term used.

| Search term | Number of videos | Number of comments collected |
| --- | --- | --- |
| Magnetotherapy | 99 | 525 |
| Bach Flowers | 98 | 691 |
| Reiki | 98 | 20633 |
| Acupuncture | 75 | 8473 |
| Energetic Therapy | 60 | 1308 |
| Naturopathy | 31 | 395 |

The table bellow summarize the number of videos and comments extracted from each used scientific term.

| Search term | Number of videos | Number of comments obtained |
| --- | --- | --- |
| Cognitive Behavioral Therapy | 83 | 6225 |
| Cancer treatment | 67 | 12227 |
| Cardiac rehabilitation | 38 | 421 |
| Sports physiotherapy | 20 | 49 |
| Neurorehabilitation | 9 | 0 |
| Endoscopic procedure | 9 | 1465 |
