# Supplementary material for: Exploring Conversational Dynamics in Scientific and Pseudoscientific Health Communities on YouTube: Process Mining and Network Analysis Study
Source: J Med Internet Res. 2026 Oct 2;28:e95273. doi: 10.2196/95273 (PMC13633066; doi:10.2196/95273)
Supplement: Supplementary file 3 [file jmir-v28-e95273-s003.docx]

The table below details the mapping from the original categories in the Madden et al. taxonomy to the simplified categories used for the zero-shot classification in this study. Categories created specifically for this study are also noted.

| **Original category or categories** | **Resulting Category** |
| --- | --- |
| Information – Make comparison | Comparison |
| Information – Request  Information – Give (video content)  Information – Give (video context)  Information – Give (general)  Advice – Recommend another video.  General conversation – Request personal information.  General conversation – Give personal information | Information Exchange |
| Advice – Request | Advice request |
| Advice – Give | Advice – Give |
| Impression – General  Impression – Positive  Impression – Negative | First impression |
| Opinion – Request | Opinion - Request |
| Opinion – Give (General)  Opinion – Give (Positive)  Opinion – Give (Negative)  Opinion – Give (Mixed) | Opinion |
| Opinion – Give (Insult) | Insult |
| Opinion – Compliment | Compliment |
| Opinion – Criticism | Criticism |
| Opinion – Tribute | Tribute |
| Opinion – Speculate | Speculate |
| Expression of personal feelings – General  Expression of personal feelings – Positive  Expression of personal feelings – Negative | Expresión de sentimientos (Expression of personal feelings) |
| General conversation – Greetings | Enhorabuena (Greetings) |
| General conversation – Thaking | Agradecimiento (Thanking) |
| General conversation – Joke | Broma (Joke) |
| General conversation – Anecdote | Anécdota (Anecdote) |
| General conversation - Express plans/desires/wishes | Planes (Plans)  Deseos (Desieres) |
| General conversation – Anticipate | Anticipación de un evento (Event anticipation) |
| Non-response comments – Unclassifiable  Non-response comments – Nonsense words/punctuation | Inclasificable (Unclassifiable) |
| Non-response comments – Spam | Spam |

**Note:** “Expression of personal feelings” was the exact label provided to the zero-shot classifier. For concision, this category is referred to as “Expression of feelings” throughout the manuscript, figures, and other tables.
