# Supplementary material for: Exploring Conversational Dynamics in Scientific and Pseudoscientific Health Communities on YouTube: Process Mining and Network Analysis Study
Source: J Med Internet Res. 2026 Oct 2;28:e95273. doi: 10.2196/95273 (PMC13633066; doi:10.2196/95273)
Supplement: Supplementary file 4 [file jmir-v28-e95273-s004.docx]

A breakdown of the category frequencies in both comment corpora is shown in the table below.

| **Category** | **Total frequency** | **Frequency in the scientific audience** | **Frequency in the pseudo-scientific audience** |
| --- | --- | --- | --- |
| Expression of feelings | 18085 | 7474 | 10611 |
| Comparison | 12456 | 5759 | 6697 |
| Compliment | 5235 | 1612 | 3623 |
| Thanking | 3984 | 660 | 3324 |
| Desires | 2071 | 289 | 1782 |
| Insult | 1817 | 497 | 1320 |
| Advice request | 1808 | 982 | 826 |
| Medical treatment | 1255 | 969 | 286 |
| Greetings | 1147 | 321 | 826 |
| Speculation | 857 | 400 | 457 |
| Advice Give | 485 | 226 | 259 |
| Joke | 478 | 158 | 320 |
| pseudo-scientific treatment | 476 | 177 | 299 |
| Criticism | 451 | 214 | 237 |
| Anecdote | 402 | 175 | 227 |
| Tribute | 368 | 73 | 295 |
| Plans | 217 | 61 | 156 |
| Unclassifiable | 216 | 92 | 124 |
| Opinion | 181 | 87 | 94 |
| Information Exchange | 113 | 52 | 61 |
| Opinion - Request | 79 | 30 | 49 |
| Emotional Support | 77 | 10 | 67 |
| Event anticipation | 63 | 27 | 36 |
| Spam | 60 | 32 | 28 |
| First impression | 30 | 9 | 21 |

**Note:** “Expression of personal feelings” was the exact label provided to the zero-shot classifier. For concision, this category is referred to as “Expression of feelings” throughout the manuscript, figures, and other tables.
