# Supplementary material for: Exploring Conversational Dynamics in Scientific and Pseudoscientific Health Communities on YouTube: Process Mining and Network Analysis Study
Source: J Med Internet Res. 2026 Oct 2;28:e95273. doi: 10.2196/95273 (PMC13633066; doi:10.2196/95273)
Supplement: Supplementary file 5 [file jmir-v28-e95273-s005.docx]

In the following table, we can see the distribution of categories assigned by the classifier to the cases that had no consensus with any human.

**Table S1.** Frequency of no-overlap cases by zero-shot thematic category

| **Classifier Category** | **Total Classifier cases** | **No human overlap, n (%)** |
| --- | --- | --- |
| **‍‍‍Expression of feelings** | 40 | 18 (45%) |
| **Comparison** | 23 | 13 (56.5%) |
| **‍‍‍Compliment** | 10 | 5 (50%) |
| **‍‍‍Desires** | 4 | 4 (100%) |
| **‍‍‍Insult** | 3 | 3 (100%) |
| **‍‍‍Advice request** | 3 | 2 (66.7%) |
| **‍‍‍Speculation** | 2 | 1 (50%) |
| **Advice Give** | 1 | 0 |
| **Greetings** | 2 | 0 |
| **Medical treatment** | 3 | 0 |
| **Thanking** | 7 | 0 |

**Note:** No human overlap indicates that neither annotator selected the thematic category assigned by the zero-shot classifier. Here, n denotes the number of comments with no human overlap. Percentages were calculated within each classifier category using the total number of classifier cases as the denominator.

As follows, is displayed how strong was the consensus of the selection of the humans in the comments classified in those categories by the classifier.

**Table S2.** Human agreement within no-overlap zero-shot thematic cases.

| **Category** | **No human overlap cases** | **Exact human agreement, n (%)** | **Partial human agreement, n (%)** | **No shared human label, n (%)** |
| --- | --- | --- | --- | --- |
| **‍Comparison** | 13 | 4 (30.8%) | 9 (69.2%) | 0 (0%) |
| **‍Expression of feelings** | 18 | 2 (11.1%) | 12 (66.7%) | 4 (22.2%) |

**Note:** No-overlap cases were comments for which neither annotator selected the zero-shot category assigned by the model. Here, n denotes the number of comments. Exact agreement indicates identical alternative label sets; partial agreement indicates at least one shared alternative label but non-identical sets; and no shared human label indicates no alternative label in common. Percentages were calculated within each zero-shot category.

**Table S3.** Most frequent human labels in no-overlap zero-shot thematic cases.

| **Zero-shot category** | **Shared human label** | **Shared label coincidences, n (%)** |
| --- | --- | --- |
| Expression of feelings | Thanking | 5 (21.7%) |
| Expression of feelings | Advice Give | 4 (17.4%) |
| Expression of feelings | Medical treatment | 4 (17.4%) |
| Expression of feelings | Compliment | 3 (13%) |
| Expression of feelings | Criticism | 3 (13%) |
| Expression of feelings | Desires | 2 (8.7%) |
| Expression of feelings | Speculation | 2 (8.7%) |
| Comparison | Advice Give | 5 (25%) |
| Comparison | Criticism | 5 (25%) |
| Comparison | Medical treatment | 3 (15%) |
| Comparison | Speculation | 2 (10%) |
| Comparison | Thanking | 2 (10%) |
| Comparison | Advice request | 1 (5%) |
| Comparison | Compliment | 1 (5%) |
| Comparison | Insult | 1 (5%) |

**Note:** No-overlap cases were comments for which neither annotator selected the category assigned by the zero-shot classifier. Here, n denotes the number of shared label coincidences. A shared label coincidence indicates that both annotators selected the same alternative label for the same comment. Percentages were calculated within each zero-shot category using the total number of shared label coincidences as the denominator. Because multiple labels could be jointly selected for the same comment, frequencies represent label coincidences rather than numbers of comments.
