# Supplementary material for: Exploring Conversational Dynamics in Scientific and Pseudoscientific Health Communities on YouTube: Process Mining and Network Analysis Study
Source: J Med Internet Res. 2026 Oct 2;28:e95273. doi: 10.2196/95273 (PMC13633066; doi:10.2196/95273)
Supplement: Supplementary file 6 [file jmir-v28-e95273-s006.docx]

**Table S1.** Summary of the main characteristics of the scientific and pseudoscientific process models

| **Characteristic** | **Pseudoscientific Proces** | **Scientific Proces** |
| --- | --- | --- |
| **‍Total initial transitions** | 4128 | 1281 |
| **‍Number of categories directly reached from the initial state** | 6 | 4 |
| **‍Most frequent initial transition** | Positive expression of feelings: 1,838 | Negative expression of feelings: 465 |
| **‍Second most frequent initial transition** | Thanking: 929 | Positive compliment: 305 |
| **‍Most frequent self-transition** | Positive expression of feelings: 630 | Positive expression of feelings: 140 |
| **‍Number of categories with a direct transition to the final state** | 6 | 9 |
| **‍Most frequent transition to the final state** | Emoji or rief acknowledgement: 1,207 | Positive expression of feelings: 358 |
| **‍Second most frequent transition to the final state**‍ | Positive expression of feelings: 1,121 | Negative expression of feelings: 212 |

### Note. Transition frequencies are reported as absolute counts. The initial and final states represent the beginning and end of a conversation trace, respectively.

#### Table S2. Normalized node-strength metrics in the scientific and pseudoscientific thematic transition networks

| Node  ‍ | Total strength | | | In-strength | | Out-Strength | |
| --- | --- | --- | --- | --- | --- | --- | --- |
|  | Pseudo | Sci | Δ (pp) | Pseudo | Sci | Pseudo | Sci |
| ‍Expression of feelings \| Negative | 8.32 | 17.51 | -9.19 | 7.39 | 15.94 | 9.26 | 19.07 |
| ‍Comparison \| Negative | 8.05 | 15.39 | -7.34 | 7.76 | 15.36 | 8.33 | 15.43 |
| ‍Thanking | 9.72 | 2.98 | 6.74 | 9.71 | 3.2 | 9.74 | 2.75 |
| ‍Expression of feelings \| Positive | 21.68 | 15.39 | 6.29 | 18.71 | 15.26 | 24.65 | 15.51 |
| Emoji-only or Brief Acknowledgement | 9.54 | 3.8 | 5.74 | 12.89 | 4.91 | 6.19 | 2.69 |
| ‍Medical treatment | 0.98 | 4.57 | -3.59 | 0.91 | 4.38 | 1.06 | 4.76 |
| ‍Compliment | 10.73 | 7.24 | 3.49 | 11.26 | 7.16 | 10.2 | 7.33 |
| ‍Advice request | 2.49 | 4.43 | -1.94 | 1.99 | 3.66 | 2.99 | 5.19 |
| ‍Comparison \| Positive | 10.65 | 8.87 | 1.78 | 10.44 | 8.93 | 10.86 | 8.81 |
| ‍Expression of feelings \| Neutral | 2.95 | 4.36 | -1.41 | 2.68 | 4.65 | 3.22 | 4.08 |
| ‍Comparison \| Neutral | 3.57 | 4.78 | -1.21 | 3.7 | 5.27 | 3.45 | 4.29 |

**Note.** Strength values are normalized and expressed as percentages of the total transition weight within each network. Total strength is calculated as the mean of normalized in-strength and out-strength. Δ represents the difference in total strength between the pseudoscientific and scientific networks, calculated as Pseudo - Sci, in percentage points. Nodes are ordered by the absolute value of Δ. The table includes the nodes with the largest between-network differences; all remaining nodes had absolute differences below 1 percentage point. Pseudo = pseudoscientific network; Sci = scientific network.
