# Supplementary material for: Exploring Conversational Dynamics in Scientific and Pseudoscientific Health Communities on YouTube: Process Mining and Network Analysis Study
Source: J Med Internet Res. 2026 Oct 2;28:e95273. doi: 10.2196/95273 (PMC13633066; doi:10.2196/95273)
Supplement: Supplementary file 7 [file jmir-v28-e95273-s007.docx]

**Table S1.** Network metrics testing different thresholds.

| **Network** | **Threshold** | **Remaining edges,**  **Percent (n/N)** | **Remaining weight,**  **Percent (n/N)** | **Top 5 nodes by weight strength** |
| --- | --- | --- | --- | --- |
| **Pseudoscientific** | 50 | 13.72% (80/583) | 77.08% (15945/20687) | Expression of feelings Positive; Comparison Positive; Compliment; Thanking; Emoji-only or Brief Acknowledgement |
| **Pseudoscientific** | 75 | 10.46% (61/583) | 71.61% (14813/20687) | Expression of feelings Positive; Compliment; Comparison Positive; Thanking; Emoji-only or Brief Acknowledgement |
| **Pseudoscientific** | 100 | 8.4% (49/583) | 66.49% (13755/20687) | Expression of feelings Positive; Compliment; Comparison Positive; Thanking; Emoji-only or Brief Acknowledgement |
| **Pseudoscientific** | 125 | 7.55% (44/583) | 63.76% (13189/20687) | Expression of feelings Positive; Compliment; Comparison Positive; Thanking; Emoji-only or Brief Acknowledgement |
| **Pseudoscientific** | 150 | 6% (35/583) | 57.96% (11990/20687) | Expression of feelings Positive; Compliment; Comparison Positive; Thanking; Emoji-only or Brief Acknowledgement |
| **Scientific** | 50 | 13.83% (74/535) | 72.25% (10538/14586) | Expression of feelings Negative; Expression of feelings Positive; Comparison Negative; Comparison Positive; Compliment |
| **Scientific** | 75 | 9.91% (53/535) | 63.73% (9296/14586) | Expression of feelings Negative; Expression of feelings Positive; Comparison Negative; Comparison Positive; Compliment |
| **Scientific** | 100 | 6.92% (37/535) | 54.37% (7931/14586) | Expression of feelings Negative; Expression of feelings Positive; Comparison Negative; Comparison Positive; Compliment |
| **Scientific** | 125 | 4.67% (25/535) | 45.4% (6622/14586) | Expression of feelings Negative; Expression of feelings Positive; Comparison Negative; Comparison Positive; Compliment |
| **Scientific** | 150 | 4.11% (22/535) | 42.64% (6219/14586) | Expression of feelings Negative; Expression of feelings Positive; Comparison Negative; Comparison Positive; Compliment |

**Figure S1.** Cumulative conversation coverage by number of retained process variants.

**Figure S2.** Results of the sensitivity analysis of the networks to the source of the comments. Points represent the absolute change in transition probability after excluding each search term. Crosses indicate transitions with at least 100 observations in the baseline network. The dashed line marks a 5-percentage-point threshold.
