# Supplementary material for: Exploring Conversational Dynamics in Scientific and Pseudoscientific Health Communities on YouTube: Process Mining and Network Analysis Study
Source: J Med Internet Res. 2026 Oct 2;28:e95273. doi: 10.2196/95273 (PMC13633066; doi:10.2196/95273)
Supplement: Supplementary file 8 [file jmir-v28-e95273-s008.docx]

**The table below presents** the probability of weight links for both networks prior to the application of any filter.

**Note:** *The X-axis (Edge Weight) is the count of how many times a specific transition occurred. The Y-axis (Weight Probability) is the proportion of all unique transitions that have that specific weight. This log-log plot shows the probability distribution of edge weights (transition counts) for both networks. The "long-tail" shape confirms that both networks are dominated by a small number of very strong ties, justifying our filtering approach to isolate this core structure.*
