# Supplementary material for: Exploring Conversational Dynamics in Scientific and Pseudoscientific Health Communities on YouTube: Process Mining and Network Analysis Study
Source: J Med Internet Res. 2026 Oct 2;28:e95273. doi: 10.2196/95273 (PMC13633066; doi:10.2196/95273)
Supplement: Supplementary file 9 [file jmir-v28-e95273-s009.docx]

| STROBE item | Item | STROBE recommendation | Location in manuscript |
| --- | --- | --- | --- |
| Title and abstract | 1 (a) | Indicate the study’s design with a commonly used term in the title or the abstract | Adressed in abstract |
|  | 1 (b) | Provide in the abstract an informative and balanced summary of what was done  and what was found | Adressed in abstract |
| **Introduction** | | | |
| Background/rationale | 2 (a) | Explain the scientific background and rationale for the investigation being reported | Addressed in the Introduction, particularly in the sections “The Proliferation of Health Misinformation on Social Media Platforms,” “Limits of Current Interventions and Analyses,” and “Process Mining and Network Analysis: Novel Methods for Understanding Conversational Dynamics” |
| Objectives | 2 (b) | State specific objectives, including any prespecified hypotheses | Addressed in the “Study Objectives” section, where the primary and secondary objectives are stated. No prespecified hypotheses were formulated because the study was exploratory and hypothesis-generating. |
| **Methods** | | | |
| Study design | 4 | Present key elements of study design early in the paper | Addressed in the Introduction, in the section “Process Mining and Network Analysis: Novel Methods for Understanding Conversational Dynamics”, where the overall methodological design is presented. |
| Setting | 5 | Describe the setting, locations, and relevant dates, including periods of recruitment, exposure, follow-up, and data collection | Addressed in the “Data Collection and Corpus Assembly” section and in the “Corpus Characteristics” section and Figure 3, where the study setting, data source, and temporal distribution of the comments are described. Recruitment, exposure, and follow-up were not applicable. |
| Participants | 6 | Participant eligibility, selection, follow-up, and matching, as applicable to cohort, case-control, or cross-sectional studies. | Not applicable. The study did not involve recruited participants or human subjects as units of analysis. It analyzed publicly available YouTube videos and comment threads; therefore, participant eligibility, follow-up, matching, and case/control selection were not applicable. |
| Variables | 7 | Clearly define all outcomes, exposures, predictors, potential confounders, and effect modifiers. Give diagnostic criteria, if applicable | Addressed in the “Comment Feature Engineering” and “Final Feature Creation” sections, where the sentiment, thematic categories, composite activities, and network variables are defined. Outcomes, exposures, predictors, confounders, effect modifiers, and diagnostic criteria were not applicable because the study was exploratory and did not estimate epidemiological associations. |
| Data sources/  measurement | 8 | For each variable of interest, give sources of data and details of methods of assessment (measurement). Describe comparability of assessment methods if there is more than one group | Addressed in the “Comment Feature Engineering,” “Sentiment Analysis,” “Thematic Categorization,” and “Final Feature Creation” sections. The same assessment methods were applied to both groups. |
| Bias | 9 | Describe any efforts to address potential sources of bias | Addressed in the “Video Classification and Filtering,” “Data Quality Control,” “Sensitivity Analyses,” and “Limitations” sections. Potential bias was addressed through manual validation by two independent annotators and sensitivity analyses. Remaining risks of selection and classification bias are acknowledged in the Limitations section. |
| Study size | 10 | Explain how the study size was arrived at | Addressed in the “Data Collection and Corpus Assembly” and “Data Quality Control” sections. No formal sample size calculation was performed because the study was exploratory. Study size was determined by retrieving up to 100 of the most-viewed videos for each search term and including all eligible comment threads after the filtering and quality-control procedures. |
| Quantitative variables | 11 | Explain how quantitative variables were handled in the analyses. If applicable, describe which groupings were chosen and why | Addressed in the “Video Classification and Filtering,” “Sentiment Analysis,” and “Thematic Categorization” sections, where the video category, comment thematic category, and comment sentiment category are defined and described. |
| Statistical methods | 12 (a) | Describe all statistical methods, including those used to control for confounding | Addressed in the “Data Quality Control” and “Data Analysis” sections, where the quantitative evaluation metrics and analytical methods are described. No adjustment for confounding was performed because the study did not estimate epidemiological associations. |
|  | 12 (b) | Describe any methods used to examine subgroups and interactions | Not applicable. No subgroup or interaction analyses were conducted; the comparison between scientific and pseudoscientific corpora was part of the primary study design. |
|  | 12 (c) | Explain how missing data were addressed | No missing-data imputation was performed. Data exclusions and quality-control procedures are described in the “Data Quality Control” section |
|  | 12 (d) | Address loss to follow-up, matching, or sampling strategy, as applicable to the study design. | Not applicable. The study was not a cohort, case-control, or conventional cross-sectional participant-based study; therefore, loss to follow-up, matching, and sampling-adjustment methods were not applicable. |
|  | 12 (e) | Describe any sensitivity analyses | Addressed in the “Sensitivity Analyses and Parameter Calibration” section. Sensitivity analyses examined the effect of different edge-weight thresholds, the number of retained process variants, and the exclusion of individual search terms. |
| **Results** | | | |
| Participants | 13 (a) | Report numbers of individuals at each stage of study—eg numbers potentially eligible, examined for eligibility, confirmed eligible, included in the study, completing follow-up, and analysed | Addressed in the “Data Collection and Corpus Assembly,” “Video Classification and Filtering,” and “Corpus Characteristics” sections, where the numbers of retrieved, included, and analyzed videos, comments, and conversation threads are reported. |
|  | 13 (b) | Give reasons for non-participation at each stage | Addressed in the “Video Classification and Filtering” and “Data Quality Control” sections, where the criteria and reasons for excluding videos and comments are described. Non-participation was not applicable because no participants were recruited. |
|  | 13 (c) | Consider use of a flow diagram | Addressed in Figures 1 and 2, which summarize the video retrieval, filtering, comment collection, and analysis processes. |
| Descriptive data | 14 (a) | Give characteristics of study participants (eg demographic, clinical, social) and information on exposures and potential confounders | Partially applicable. Addressed in the “Corpus Characteristics” section, where the characteristics of the analyzed videos and comments, including language, publication period, and thematic distribution, are reported. Participant demographics, exposures, and confounders were not applicable. |
|  | 14 (b) | Indicate number of participants with missing data for each variable of interest | Not applicable. No participant-level variables were collected. Data exclusions and quality-control procedures are described in the “Data Quality Control” section |
|  | 14 (c) | Cohort study—Summarise follow-up time (eg, average and total amount) | Not applicable. This was not a cohort study and no participant follow-up was conducted. |
| Outcome data | 15 | Report outcome events or summary measures, as applicable to the study design. | Partially applicable. Addressed in the “Corpus Characteristics,” “Process Models of Audience Interaction,” and “Thematic Transition Networks” sections, where summary measures and the main analytical results are reported separately for the scientific and pseudoscientific corpora. Conventional outcome events or exposure measures were not applicable because the study did not evaluate epidemiological outcomes. |
| Main results | 16 (a) | Give unadjusted estimates and, if applicable, confounder-adjusted estimates and their precision (eg, 95% confidence interval). Make clear which confounders were adjusted for and why they were included | Partially applicable. Descriptive estimates and summary measures are reported in the Results section. Adjusted estimates and confidence intervals were not calculated because the study did not assess epidemiological associations or control for confounders. |
|  | 16 (b) | Report category boundaries when continuous variables were categorized | Addressed in the “Sentiment Analysis” section, where scores of 1–2 were categorized as negative, 3 as neutral, and 4–5 as positive. |
|  | 16 (c) | If relevant, consider translating estimates of relative risk into absolute risk for a meaningful time period | Not applicable. The study did not estimate relative or absolute risks. |
| Other analyses | 17 | Report other analyses done—eg analyses of subgroups and interactions, and sensitivity analyses | Addressed in the “Sensitivity Analyses and Parameter Calibration” section, where additional analyses evaluating different edge-weight thresholds, numbers of retained process variants, and the exclusion of individual search terms are reported. No subgroup or interaction analyses were conducted. |
| **Discussion** | | | |
| Key results | 18 | Summarise key results with reference to study objectives | Addressed in the “Principal Results” section, where the main findings are summarized in relation to the study objectives and the comparison between scientific and pseudoscientific conversational patterns. |
| Limitations | 19 | Discuss limitations of the study, taking into account sources of potential bias or imprecision.  Discuss both direction and magnitude of any potential bias | Addressed in the “Limitations” section, where potential selection, classification, language, and platform-related biases are discussed. Their possible magnitude is described where quantitative evidence was available, including the model-validation and sensitivity-analysis results. For other potential biases, the direction and magnitude could not be reliably quantified and are explicitly acknowledged. |
| Interpretation | 20 | Give a cautious overall interpretation of results considering objectives, limitations, multiplicity of analyses, results from similar studies, and other relevant evidence | Addressed in the “Principal Results,” “Comparison with Prior Work,” and “Limitations” sections, where the findings are interpreted cautiously in relation to the study objectives, previous research, and the methodological limitations. The conclusions are presented as exploratory and hypothesis-generating rather than definitive. |
| Generalisability | 21 | Discuss the generalisability (external validity) of the study results | Addressed in the “Limitations” section, where the generalisability of the findings is discussed. |
| **Other information** | | | |
| Funding | 22 | Give the source of funding and the role of the funders for the present study and, if applicable,  for the original study on which the present article is based | Addressed in the “Funding” section. The funding source is reported, and the role of the funder has been clarified. |
